# Antigen-specific decidual CD8^+^ T cells include distinct effector memory and tissue-resident memory cells

**DOI:** 10.1172/jci.insight.171806

**Published:** 2023-09-08

**Authors:** Shweta Mahajan, Aria Alexander, Zachary Koenig, Nicholas Saba, Nina Prasanphanich, David A. Hildeman, Claire A. Chougnet, Emily DeFranco, Sandra Andorf, Tamara Tilburgs

**Affiliations:** 1 Immunology Graduate Program, University of Cincinnati College of Medicine, Cincinnati, Ohio, USA.; 2 Division of Immunobiology,; 3 Division of Biomedical Informatics, and; 4 Division of Infectious disease, Cincinnati Children’s Hospital, Cincinnati, Ohio, USA.; 5 Department of Pediatrics, and; 6 Department of Obstetrics and Gynecology, University of Cincinnati College of Medicine, Cincinnati, Ohio, USA.; 7 Division of Allergy & Immunology, and; 8 Center for Inflammation and Tolerance, Cincinnati Children’s Hospital Medical Center, Cincinnati, Ohio, USA.

**Keywords:** Immunology, Reproductive Biology, Reproductive biochemistry, T cells

## Abstract

Maternal decidual CD8^+^ T cells must integrate the antithetical demands of providing immunity to infection while maintaining immune tolerance for fetal and placental antigens. Human decidual CD8^+^ T cells were shown to be highly differentiated memory T cells with mixed signatures of dysfunction, activation, and effector function. However, no information is present on how specificity for microbial or fetal antigens relates to their function or dysfunction. In addition, a key question, whether decidual CD8^+^ T cells include unique tissue-resident memory T cells (Trm) or also effector memory T cell (Tem) types shared with peripheral blood populations, is unknown. Here, high-dimensional flow cytometry of decidual and blood CD8^+^ T cells identified 2 Tem populations shared in blood and decidua and 9 functionally distinct Trm clusters uniquely found in decidua. Interestingly, fetus- and virus-specific decidual CD8^+^ Trm cells had similar features of inhibition and cytotoxicity, with no significant differences in their expression of activation, inhibitory, and cytotoxic molecules, suggesting that not all fetus-specific CD8^+^ T cell responses are suppressed at the maternal-fetal interface. Understanding how decidual CD8^+^ T cell specificity relates to their function and tissue residency is crucial in advancing understanding of their contribution to placental inflammation and control of congenital infections.

## Graphical abstract

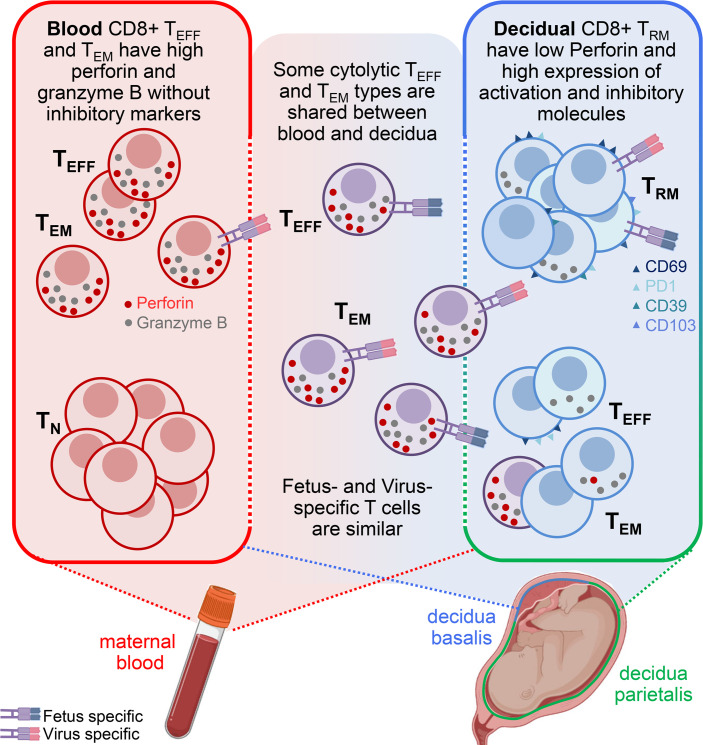

## Introduction

To establish and maintain a healthy pregnancy, the maternal immune system must tolerate fetal and placental alloantigens and have strong capacity to clear infections (1). Maternal decidual CD8^+^ T cells found at the maternal-fetal interface in the placenta are key immune effector cells that can directly recognize and respond to fetal and viral antigens (2). Clinically, infiltrating and clonally expanded CD8^+^ T cells are observed during chronic inflammation of the placental membranes (chorioamnionitis) and of the placental villi (villitis) (3–7). While chronic chorioamnionitis and villitis are observed during placental infections, both are frequently found in the absence of an infective etiology (3, 4, 8). This has prompted speculation of a breakdown of maternal-fetal tolerance and increased CD8^+^ cytotoxic T cell (CTL) responses to foreign fetal alloantigens that may resemble transplant rejection (3, 4). Decidual CD8^+^ T cells form approximately 7% of CD45^+^ leukocytes in first-trimester decidua and their proportion increases significantly to approximately 30%–50% of lymphocytes in decidua at term pregnancy (9, 10). Our previous studies demonstrated that the majority of decidual CD8^+^ T cells in first trimester and term pregnancy are differentiated memory T cells, and compared with blood CD8^+^ effector memory T (Tem) cells have increased protein and RNA signatures of dysfunction, activation, and effector function (11, 12). These paradoxical features may provide temporary CD8^+^ T cell inactivation of fetus-specific T cells at the maternal-fetal interface to allow trophoblast invasion and placental growth, while other decidual CD8^+^ T cell types retain capacity to respond to infection. However, numerous gaps remain in the understanding of how decidual CD8^+^ T cells are regulated during healthy pregnancy. It prompts the question whether separately regulated decidual CD8^+^ tissue-resident memory T cell (Trm) populations, including hyporesponsive fetus-specific and activated cytolytic microbe-specific T cells, are present that can simultaneously accommodate maternal-fetal tolerance and placental immunity.

Unique features of decidual CD8^+^ memory T cells include the absence of perforin (PFN) protein expression and the significant reduction in granzyme B (GZMB) protein expression compared with peripheral blood CD8^+^ Tem cells (11–14). Furthermore, decidual CD8^+^ T cells express increased levels of coinhibitory markers, including CD39, programmed cell death-1 (PD1), and T cytotoxic T lymphocyte–associated protein 4 (CTLA4) in combination with increased expression of T cell activation markers, including CD25, inducible T cell costimulator (ICOS), glucocorticoid-induced TNF receptor family–related protein (GITR), markers for tissue residency (CD69 and CD103) (15–17), and the antimicrobial peptide granulysin (GNLY) compared with blood (11–14). Upon in vitro stimulation, decidual CD8^+^ T cells upregulated PFN and GZMB expression, secreted IFN-γ and TNF-α, and degranulated at levels equal or higher compared to peripheral blood CD8^+^ Tem cells (12). While these observations informed us on the differences between blood and decidual CD8^+^ T cells, they did not investigate the likely phenotypic and functional diversity of decidual CD8^+^ Tem and Trm cell types in detail.

CD8^+^ T cell activation and differentiation into T effector (Teff) and Tem cells includes the gain of IFN-γ and TNF-α production and a significant increase in cytolytic capacity. In contrast, T cell dysfunction is characterized by loss of IL-2, IFN-γ, and TNF-α production combined with diminished T cell survival, reduced proliferative capacity, and low T cell cytotoxicity (18–21). Dysfunctional T cells include exhausted CD8^+^ T cells that initially obtain effector functions and become dysfunctional during chronic exposure to antigen as well as anergic T cells that fail to gain effector functions due to priming without costimulation (18, 20, 22–24). T cells may also be temporarily inhibited in their effector functions after interaction with immunosuppressive cells such as regulatory T cells (Tregs) and become dysfunctional (18, 25). Recently, we demonstrated that decidual CD8^+^ T cells had reduced proliferation, reduced production of IFN-γ and TNF-α, and increased IL-10 production upon coculture with decidual Tregs from the same placental sample (25). Other types of CD8^+^ Tregs with a suppressive function have also been described (26–28). A variety of markers have been implicated to separate dysfunctional from activated T cells, but oftentimes the expression of inhibitory molecules is not exclusive to dysfunctional T cells, as they are also observed on activated T cells (19, 22, 23, 29, 30). The significant overlap of gene expression profiles and cell-surface markers between dysfunctional and activated T cells makes functional assessment of CD8^+^ T cell types, including cytokine secretion, survival capacity, and cytotoxicity, necessary to separate distinct functional and dysfunctional T cell types.

Another important question is how CD8^+^ T cell function and dysfunction are related to their antigen specificity. During pregnancy, fully functional virus-specific CD8^+^ Teff cells are needed to maintain immunity to placental infection, while simultaneously fetus-specific CD8^+^ Teff cells need regulation to prevent detrimental allogeneic responses to fetal alloantigens expressed by placental trophoblasts (1, 31). Fetus-specific CD8^+^ T cell responses to Y chromosome–derived residues (referred to as HY) are frequently found in blood and decidua of healthy mothers carrying male offspring (2, 32–34). HY-specific CD8^+^ T cells were found in 50% of mothers pregnant with a first boy, and up to 75% of woman that had more than 2 boys. While the function of these cells in decidua is unknown, in blood, both HY-specific CD8^+^ T cells that secreted IFN-γ and directly lysed male target cells as well as hyporesponsive HY-specific CD8^+^ T cells were identified (33, 34). Virus-specific T cell responses are also effectively generated during pregnancy and increased percentages of virus-specific CD8^+^ T cells were found in decidual tissue compared with blood after uncomplicated term pregnancy (35). But interestingly, human cytomegalovirus–specific (HCMV-specific) CD8^+^ T cells in decidua had reduced levels of PFN compared with HCMV-specific CD8^+^ T cells in blood, suggesting the decidual environment influences the cytotoxic capacity of decidual virus-specific CD8^+^ T cells (12). However, no detailed description of how specificity for fetal and viral antigens relates to the decidual CD8^+^ T cell phenotype and function has been presented thus far.

This study investigates CD8^+^ T cells purified from term pregnancy decidua basalis, the decidua lining the placental villi at the implantation site, as well as decidua parietalis, the decidual tissue lining the chorionic membrane (11, 12). These sites both have clinical relevance for development of placental inflammation, including deciduitis, villitis, and chorioamnionitis (3–5, 36, 37). We utilized high-dimensional spectral flow cytometry, MHC/peptide dextramer staining, and computational analysis combined with in-depth functional analysis of T cell survival, degranulation, and cytokine secretion and identified phenotypically and functionally distinct decidual CD8^+^ Tem and Trm cell types. Understanding how decidual CD8^+^ T cell specificity relates to their function and tissue residency is crucial in advancing understanding of their contribution to placental inflammation and control of congenital infections.

## Results

### Decidual CD8^+^ T cells contain distinct Trm clusters not found in peripheral blood.

**Figure 1:**
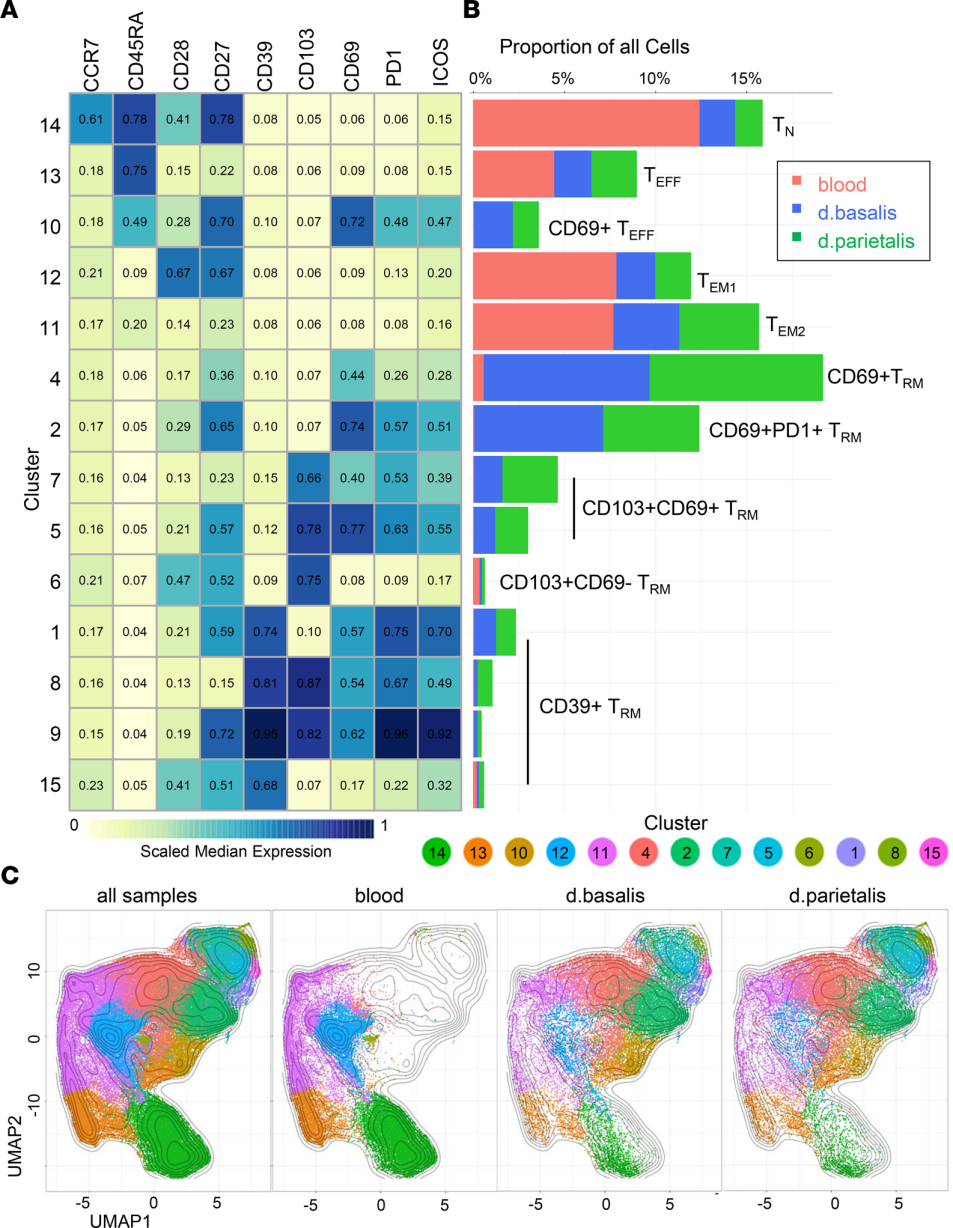
FlowSOM identifies phenotypically distinct blood and decidual CD8^+^ T cell clusters. FlowSOM populations were generated based on expression profiles of CD45^+^CD14^–^CD56^–^CD3^+^CD4^–^CD8^+^ T cells from blood (*n* = 5), decidua basalis (*n* = 5), and decidua parietalis (*n* = 5). (**A**) Heatmap shows the arcsinh-transformed scaled median fluorescence intensity (MFI) of the specified markers for the 14 FlowSOM populations. (**B**) Graph depicts the frequency of cells in each cluster as percentage of all analyzed cells. (**C**) Uniform manifold approximation and projection (UMAP) graphs depict CD8^+^ T cell cluster distribution in all samples combined as well as those separated by blood, decidua basalis, and decidua parietalis.

**Figure 2:**
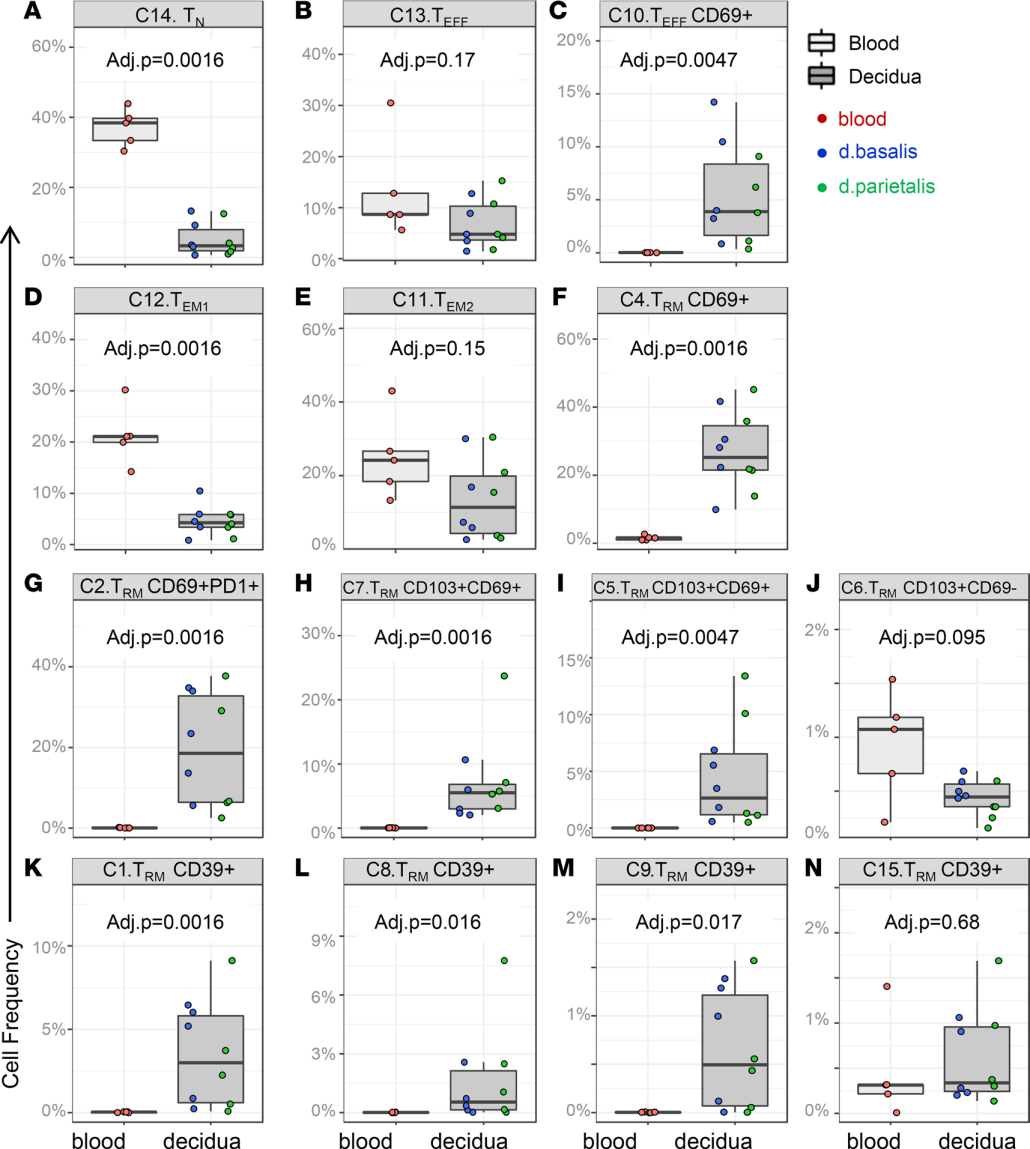
Cell frequency per cluster is significantly different between blood and decidua. (**A**–**N**) Graphs depict cell frequencies per cluster in blood and decidual tissues. Boxes depict median and IQR in all blood (*n* = 5) (light gray boxes) compared with combined decidua basalis (*n* = 5) and decidua parietalis (*n* = 5) (dark gray boxes. *P* values by Wilcoxon’s rank-sum test and FDR-adjusted.

To determine decidual CD8^+^ T cell diversity, paired lymphocyte isolates of decidua basalis and decidua parietalis obtained after healthy term pregnancy (gestational age 37–42 weeks) were stained with a 21-parameter high-dimensional spectral flow cytometry panel and compared to peripheral blood obtained from healthy nonpregnant donors (Supplemental Table 1; supplemental material available online with this article; https://doi.org/10.1172/jci.insight.171806DS1). Technical controls were included in each batch to identify possible batch effects (38). The panel included markers used for gating (CD45, CD14, CD3, CD56, CD4, and CD8), CD8^+^ T cell differentiation markers (CD45RA, CCR7, CD28, and CD27), cytolytic granules (PFN, GZMB, and GNLY), coinhibitory molecules (PD1, CD39, and CTLA4), activation markers (ICOS, CD25, and GITR), and markers for tissue residency (CD69 and CD103), which were selected based on previous RNA and protein expression profiles of decidual CD8^+^ T cells (11–14). CD8^+^ T cells were gated using the strategy depicted in Supplemental Figure 1A, and multidimensional scaling (MDS) plots were generated to determine the similarity between CD8^+^ T cell samples. MDS plots separated blood and decidual samples on the first dimension (Supplemental Figure 2A), but no batch-related separation of the CD8^+^ T cell samples was observed (Supplemental Figure 2B). Next, a principal component analysis–based (PCA-based) nonredundancy score (NRS) plot was generated to visualize the contribution of each of the analyzed parameters to CD8^+^ T cell diversity (Supplemental Figure 3A) (39). Parameters with a high NRS score (e.g., CD45RA, CD69, and PD1) explain more of the diversity observed in each sample. Three markers, GITR, CD25, and CTLA4, had the lowest NRS scores and had virtually no expression on blood or decidual CD8^+^ T cells and were excluded from further analysis (Supplemental Figure 3, A and B). Next high-dimensional clustering analysis using the flow self-organizing maps (FlowSOM) algorithm (40) was performed using the expression of the 9 remaining cell-surface markers on blood, decidua basalis, and decidua parietalis CD8^+^ T cells. Of the 15 clusters generated, 2 clusters, cluster 1 and cluster 3, were relatively small (0.79% and 1.52%, respectively) and had only minor differences in the expression of the analyzed parameters (Supplemental Figure 4, A and B). Therefore, clusters 1 and 3 were merged and from here on referred to as cluster 1 (Figure 1A). The proportions and the expression profiles of the 14 clusters are depicted in Figure 1, A and B, and Supplemental Figure 5. Importantly each of the generated clusters included cells from multiple donors (Figure 2). While previous studies found many differences in Treg and decidual NK (dNK) types and frequencies between decidual basalis and decidua parietalis (25, 41, 42), there were no significant differences in the CD8^+^ T cell clusters between decidua basalis and decidua parietalis, suggesting that CD8^+^ T cells have similar roles at these 2 sites (Supplemental Figure 6A). In contrast, many clusters had significant differences in cell frequency between blood and decidua (Figure 2). The FlowSOM clustering identified (i) 1 cluster of CCR7^+^CD45RA^+^) naive CD8^+^ T cells (Tn), which were highly enriched in blood (Figure 1B and Figure 2A); (ii) 2 clusters of CD8^+^ Teff cells (CCR7^–^CD45RA^+^), of which cluster 13 was found in both blood and decidua and cluster 10 only in decidua (Figure 2, B and C). Phenotypically, cluster 10 had increased expression of CD27, CD69, PD1, and ICOS compared with cluster 13, suggesting increased activation of this decidual Teff cell type (Figure 1A); (iii) 2 clusters of CCR7^–^CD45RA^–^ CD8^+^ Tem cells that were present in both blood and decidua (clusters 11 and 12) (Figure 1A and Figure 2, D and E); and (iv) 9 clusters of CCR7^–^CD45RA^–^ CD8^+^ Trm cells identified by the expression of CD103 and/or CD69 (15–17). Trm were predominantly found in decidua and had a high phenotypic diversity in their expression of T cell differentiation (CD27, CD28, CD103), activation (CD69, ICOS) and inhibitory (CD39, PD1) markers (Figure 1 and Figure 2, F–N). This demonstrates that decidual CD8^+^ T cells are very heterogeneous and contain Teff and Tem types that are also found in blood, while having several additional CD8^+^ Trm types that are unique to decidua and not found in blood. A direct comparison of peripheral blood CD8^+^ T cells from nonpregnant controls with maternal peripheral blood showed no significant differences in the frequencies of the CD8^+^ Tn, Tcm, Teff, and Tem populations (Supplemental Figure 6B), the percentage of CD39^+^, CD103^+^, CD69^+^, or PD1^+^ CD8+ T cells (Supplemental Figure 6C), or the percentage PFN^+^ and GZMB^+^ CD8^+^ T cells (Supplemental Figure 6, D and E), confirming previous studies (11).

### Decidual and blood CD8^+^ cell clusters have distinct expression of cytolytic granules.

**Figure 3:**
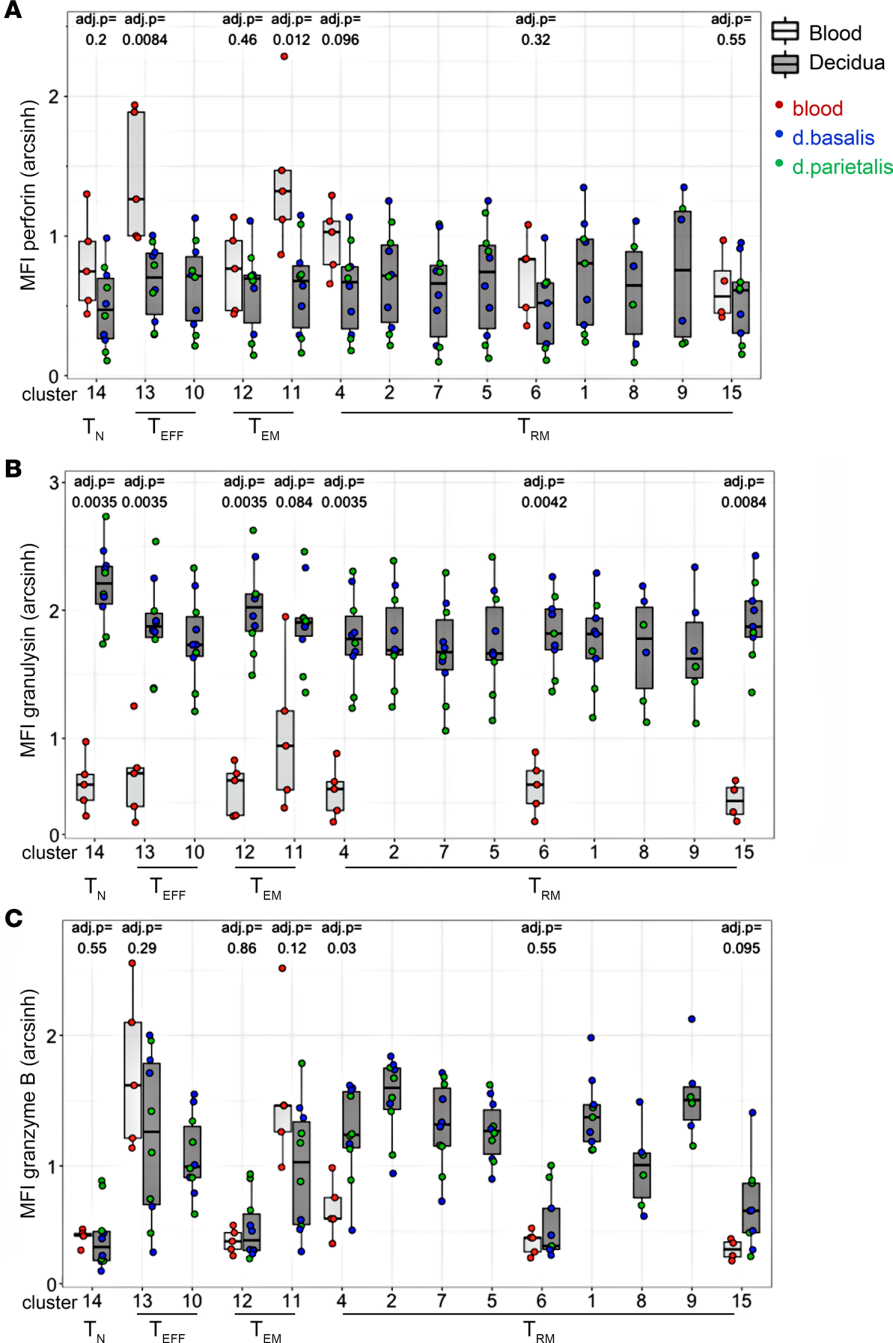
Blood and decidual CD8^+^ T cell clusters have distinct expression of cytolytic granules. Graphs depict the arcsinh-transformed median florescence intensity (MFI) of (**A**) perforin, (**B**) granulysin, and (**C**) granzyme B in all blood (*n* = 5) (light gray boxes) and decidua basalis (*n* = 5) and decidua parietalis (*n* = 5) (dark gray boxes) CD8^+^ T cell clusters. Boxes depict median and IQR. *P* values by Wilcoxon’s rank-sum test and FDR-adjusted.

Next, to assess the cytolytic potential of CD8^+^ T cell clusters in blood and decidua, the median fluorescence intensity (MFI) of intracellular PFN, GZMB, and GNLY expression as well as the frequency of PFN^+^, GZMB^+^, and GNLY^+^ cells were determined within each cluster separated by their tissue (decidua) or blood origin. MFIs and frequencies were only calculated from samples that had more than 100 cells (events) in a cluster. As expected, PFN expression was highest in blood Teff cells (cluster 13) and blood Tem cluster 11 (Figure 3A and Supplemental Figure 7A). All other CD8^+^ T cell clusters in blood and decidual tissue did not express high levels of PFN, confirming previous studies (11, 12, 43). GNLY was expressed in all decidual CD8^+^ T cell clusters, but with no significant differences between the clusters in both MFI and frequency (Figure 3B and Supplemental Figure 7B). Blood Teff cluster 13 and Tem cluster 11 had high GNLY MFI and frequencies in 3 out of the 5 samples analyzed. Of the 3 cytotoxic molecules, GZMB expression was the most variable between CD8^+^ T cell clusters. As expected and similar to PFN expression, blood Teff cluster 13 and Tem cluster 11 had the highest GZMB expression levels and frequencies of GZMB^+^ cells (Figure 3C and Supplemental Figure 7C). In decidua, both Teff clusters 13 and 10, Tem cluster 11, and the majority of Trm clusters expressed GZMB (Figure 3C). The notable differences in GZMB expression between the phenotypically distinct decidual Tem clusters suggest the decidual Tem and Trm populations are heterogeneous in phenotype and cytolytic capacity. Furthermore, the decreased PFN expression and increased GNLY expression in decidual clusters compared with phenotypically matched clusters in blood suggests that decidual CD8^+^ T cell differentiation and function is dependent on the local decidual tissue environment.

### Decidual virus-specific CD8^+^ T cells include unique Trm cells not found in blood.

**Figure 4:**
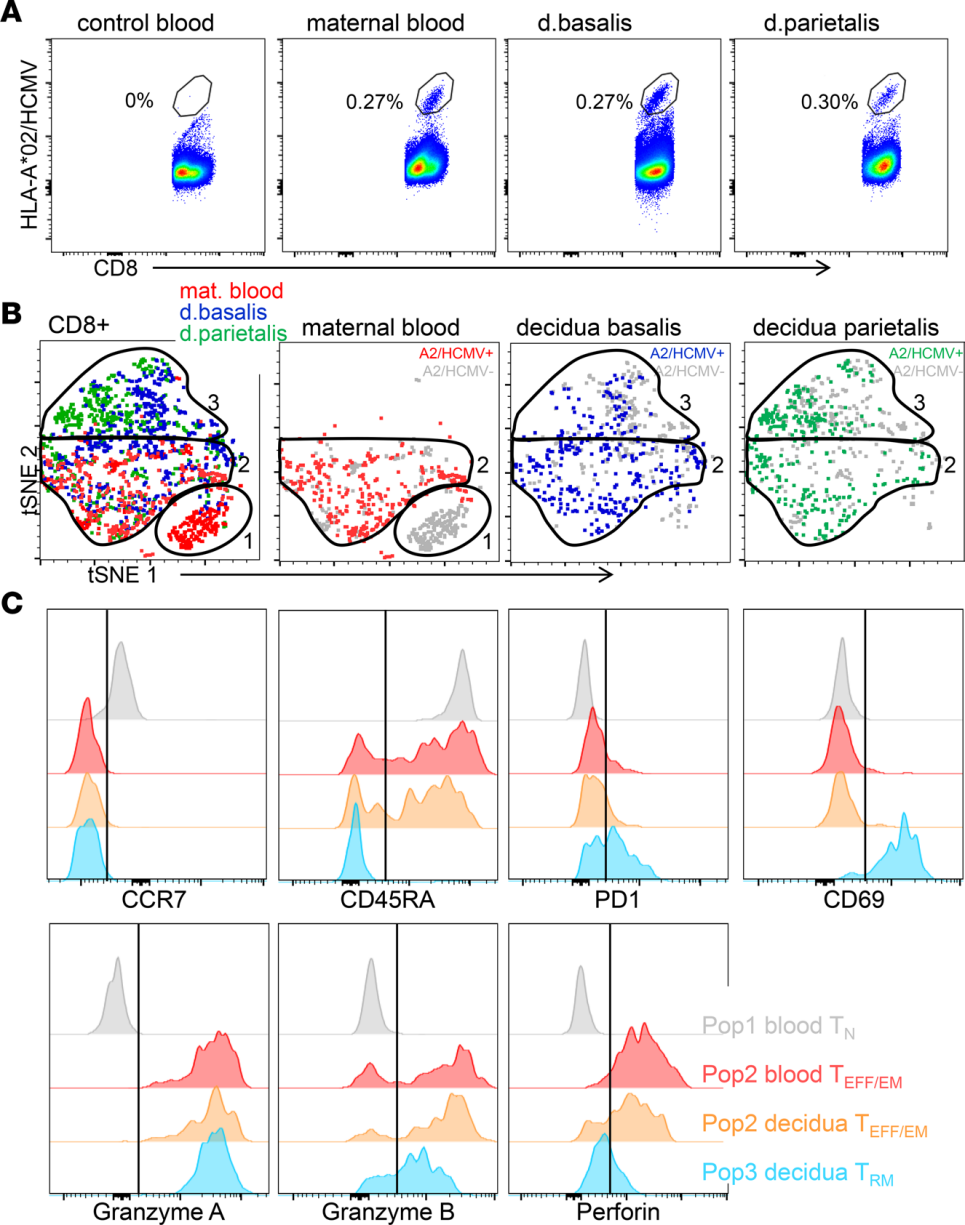
Virus-specific CD8^+^ T cells are distinct in blood and decidua. (**A**) Dot plots of CD8 and HLA-A2/HCMV dextramer staining on CD45^+^CD14^–^CD56^–^CD4^–^CD8^+^ T cells in maternal blood, decidua basalis, and decidua parietalis tissue of 1 HLA-A2^+^ donor, compared with an HLA-A2^–^ control donor. (**B**) tSNE plots of HLA-A2/HCMV–specific cells in maternal blood (red), decidua basalis (blue), and decidua parietalis (green) compared with dextramer-negative cells (gray). (**C**) Histograms of CCR7, CD45RA, PD1, CD39, granzyme A, granzyme B, and perforin expression in CD8^+^ T cell blood population 1 (gray), blood population 2 (red), decidual population 2 (orange), and decidual population 3 (blue) as gated in **B**.

Next, additional paired samples of maternal peripheral blood, decidua basalis, and decidua parietalis of an HLA-A2^+^ donor was stained with an HLA-A2/HCMV dextramer to detect virus-specific CD8^+^ T cells in maternal peripheral blood and decidual tissues of the same donor (Figure 4A). The phenotype and cytolytic function of HLA-A2/HCMV–specific CD8^+^ T cells was determined using additional staining for CCR7, CD45RA, PD1, CD69, GZMs, and PFN. First, to visualize this high-dimensional data, t-distributed stochastic neighbor embedding (t-SNE) plots were generated on dextramer-positive and -negative cells for each tissue (Figure 4B). In maternal blood, dextramer-negative CD8^+^ T cells mainly consisted of CCR7^+^CD45RA^+^ Tn cells (population 1), whereas A2/HCMV-specific cells (blood population 2) consisted of CCR7^–^CD45RA^+^ Teff and CCR7^–^CD45RA^–^ Tem cells with high expression of cytolytic molecules GZMA, GZMB, and PFN (Figure 4C). Decidual dextramer-negative CD8^+^ T cells had few (3%–5%) CCR7^+^CD45RA^+^ Tn cells (population 1, Supplemental Figure 8), showing limited contamination with peripheral blood T cells of the decidual isolates. Interestingly, about half of the A2/HCMV-specific cells in decidua basalis and decidua parietalis overlapped with maternal blood A2/HCMV-specific cells (decidua population 2). Population 2 corresponds to Teff cluster 13 and Tem clusters 11 and 12, as shown in Figure 1. However, decidual A2/HCMV^+^ cells also included an additional population of CCR7^–^CD45RA^–^ T cells (population 3), which had high levels of PD1 and CD69 but reduced levels of GZMB and PFN (Figure 4, B and C), corresponding to the Trm cells shown in Figure 1. Thus, decidual virus-specific CD8^+^ T cells include 2 populations: (i) a PFN^hi^ cytolytic CD8^+^ Teff/Tem cell type (population 2) also found in maternal blood and (ii) an additional, more dysfunctional PD1^+^CD69^+^PFN^lo^ Trm cell type (population 3) (Figure 4 and Supplemental Figure 8).

### Decidual virus- and fetus-specific CD8^+^ Tem cells are similar.

**Figure 5:**
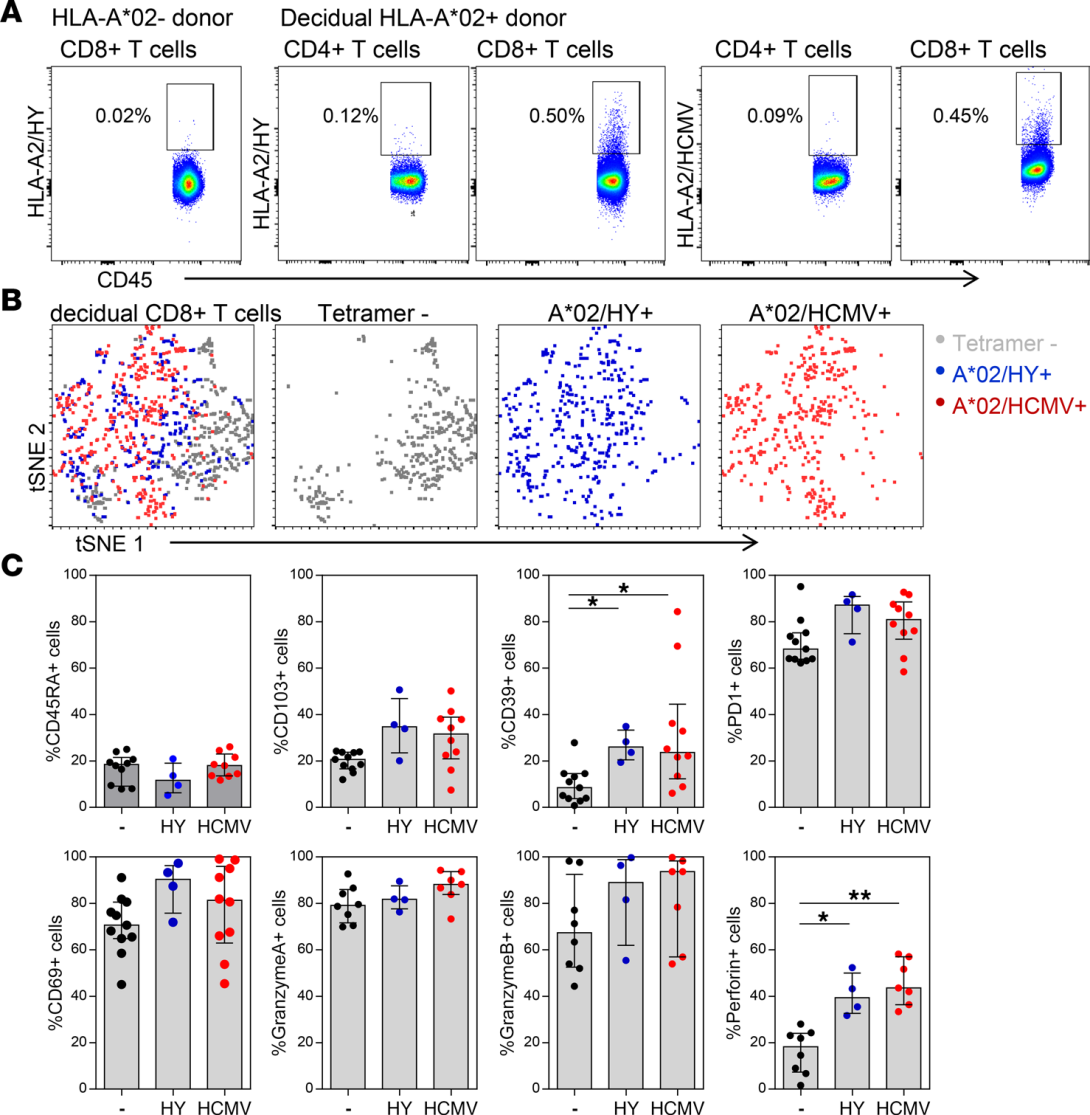
Decidual virus- and fetus-specific CD8^+^ Tem cells have similar features of inhibition and cytotoxicity. (**A**) Dot plots of CD8 and HLA-A2/HY or HLA-A2/HCMV dextramer staining on decidual CD45^+^CD14^–^CD56^–^CD4^–^CD8^+^ T cells compared with decidual CD45^+^CD14^–^CD56^–^CD8^–^CD4^+^ T cells in the same sample and an additional HLA-A2^–^ control donor. (**B**) tSNE plots of HLA-A2/HY– (blue) and HLA-A2/HCMV–specific (red) decidual CD8^+^ T cells compared with dextramer-negative cells (gray). (**C**) Graphs depict the percentage of CD45RA^+^, CD103^+^, CD39^+^, PD1^+^, CD69^+^, granzyme A^+^, granzyme B^+^, and perforin^+^ cells in HLA-A2/HY– (blue) (*n* = 4) and HLA-A2/HCMV–specific (red) (*n* = 10) decidual CD8^+^ T cells compared with dextramer-negative cells (black). Bars and lines indicate median and IQR. *P <0.05; **P <0.01 by nonparametric Kruskal-Wallis test with Dunn’s multiple-comparison post hoc test.

Next, to investigate whether CD8^+^ T cells with specificity for fetal or viral antigens are differentially regulated in decidual tissue, additional samples were stained with MHC/peptide dextramers for HLA-A2/HY and HLA-A2/HCMV in combination with staining for CCR7, CD45RA, PD1, CD69, CD39, CD103, GZMs, and PFN. Dextramer-positive CD8^+^ cells were determined in comparison to dextramer background staining on HLA-A*02^–^ donors as well as CD4^+^ T cells of the same donors (Figure 5A and Supplemental Figure 9, A–C). One placenta collected after a pregnancy with a male fetus had both HLA-A2/HY– and HLA-A2/HCMV–specific decidual CD8^+^ T cells (Figure 5A). t-SNE plots show that both these HLA-A2/HY– and HLA-A2/HCMV–specific T cells were somewhat separated from dextramer-negative cells, but that there was no clear difference between HLA-A2/HY– and HLA-A2/HCMV–specific CD8^+^ T cells (Figure 5B and Supplemental Figure 9D). Investigation of additional donors confirmed the differences between dextramer-negative CD8^+^ T cells compared with HLA-A2/HY– and HLA-A2/HCMV–specific CD8^+^ T cells, which had significantly increased frequencies of CD39^+^ and PFN^+^ cells (Figure 5C). While the frequency of PFN^+^ cells in the dextramer-negative cells is in line with the frequency of PFN^+^ cells in all clusters, the frequency of PFN^+^ HLA-A2/HY– and HLA-A2/HCMV–specific CD8^+^ T cells is higher than decidual clusters and more comparable to blood Tem clusters 11 and 4 (Figure 5C and Supplemental Figure 7). The high frequencies of CD69^+^ and PD1^+^ on the dextramer-positive cells also show these cells are not contamination from peripheral blood. Interestingly, this also confirmed the lack of differences between HLA-A2/HY^+^ and HLA-A2/HCMV^+^ cells in the expression of markers of activation, inhibition, and cytotoxicity, suggesting that fetus- and virus-specific CD8^+^ T cells are not differentially regulated in decidual tissues. The increased levels of PFN in both fetus- and virus-specific CD8^+^ T cells suggests that not all fetus-specific CD8^+^ T cell responses are suppressed at the maternal-fetal interface.

### Decidual CD8^+^ Teff, Tem, and Trm clusters have distinct secretomes.

**Figure 6:**
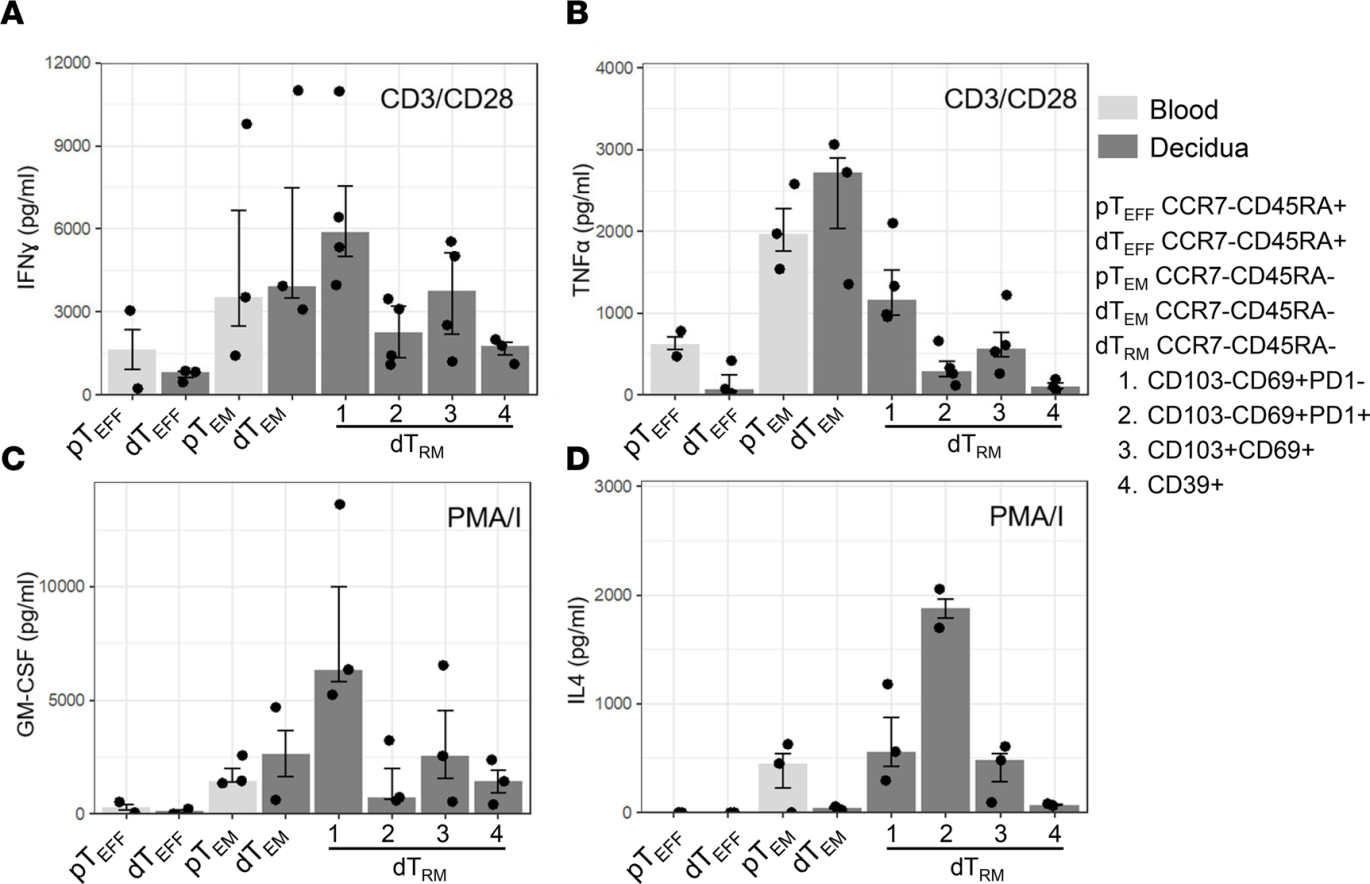
Decidual CD8^+^ Tem clusters have distinct secretomes. Graphs depict the concentration of (**A**) IFN-γ, (**B**) TNF-α, (**C**) GM-CSF, and (**D**) IL-4 in CD8^+^ T cell culture supernatants upon CD3/CD28 (20 hours) or PMA/I (6 hours) stimulation (as indicated), as determined by Isoplexis human adaptive immune secretome chip analysis. Bars depict median and IQR of *n* = 2–4 donors.

Next, to assess whether blood and decidual CD8^+^ Teff, Tem, and Trm cells have distinct functions, their cytokine secretion capacity was determined. Peripheral blood CD8^+^ T cells were sorted into CCR7^–^CD45RA^+^ Teff (pTeff) and CCR7^–^CD45RA^–^ Tem (pTem) cells, while decidual CD8^+^ T cells were sorted into CCR7^–^CD45RA^+^ Teff (dTeff), CCR7^–^CD45RA^–^Tem (dTem), and 4 types of CCR7^–^CD45RA^–^ Trm (dTrm) (Supplemental Figure 1B). Due to limitations in decidual CD8^+^ T cell yields and the small cluster frequencies found, decidual CD8^+^ Trm clusters were separated based on (i) cells that expressed CD39, indicative of a more dysfunctional phenotype (dTrm4; combined clusters 1, 8, 9, and 15); (ii) cells that expressed CD103 and CD69 but no CD39 (dTrm3; combined clusters 5 and 7); (iii) cells that expressed CD69 but not PD1, CD103, and CD39 (drm1; cluster 4); and (v) cells that expressed CD69 and PD1, but not CD103 and CD39 (dTrm2; cluster 2) according to the gating strategy depicted in Supplemental Figure 1C and Supplemental Table 2. ICOS was not included in the sorting strategy, as it closely followed PD1 expression but did not have a clear separation of negative and positive cells (Figure 1A and Supplemental Figure 5). Blood and decidual CD8^+^ T cell types were plated and stimulated with anti-CD3/anti-CD28 for 20 hours or with PMA and ionomycin (PMA/I) for 6 hours, representing both an antigen-specific TCR stimulation (CD3/CD28) and a general pan T cell stimulation (PMA/I). Supernatants were analyzed using Isoplexis human adaptive immune secretome chips, detecting 20 cytokines, PFN, and GZMB. While pTeff and dTeff as well as pTem and dTem had similar cytokine secretion profiles in direct comparison, the 4 dTrm cell types analyzed had some variation in their cytokine secretion profiles (Figure 6, A–D). Here, the CD39^+^ dTrm4 cells had low secretion of cytokines (IFN-γ, TNF-α, IL-4, and GM-CSF), while the CD39^–^CD103^–^CD69^+^PD1^–^ dTrm1 cells secreted high levels of IFN-γ, TNF-α, and GMCSF upon stimulation (Figure 6, A–C). Interestingly, PMA/I stimulation induced high IL-4 secretion in the CD39^–^CD103^–^CD69^+^PD1^+^ dTrm2 cluster but not in other populations (Figure 6D). This function was previously described for CD8^+^ Tregs as well as cytolytic CD8^+^ Tem cells (44, 45). No clear differences in IFN-γ and TNF-α secretion between T cell types were observed in response to PMA/I, possibly due to the very strong general activation that maximized the IFN-γ and TNF-α responses (Supplemental Figure 10, A and B). MIP1α and MIP1β were expressed at high levels by all blood and decidual CD8^+^ T cell types upon both CD3/CD28 and PMA/I stimulation, but interestingly the dTeff cells secreted the lowest levels (Supplemental Figure 10, C–F). Secretion of IL-13 and IL-17A was overall low and no differential expression was observed (Supplemental Figure 10, G and H). IL-5, IL-6, IL-7, IL-8, IL-9, IL-10, IL-15, IP10, MCP1, sCD137, and TNF-β were not expressed by any of the CD8^+^ T cell clusters analyzed after either CD3/CD28 or PMA/I stimulation. Overall, these data show that decidual CD8^+^ Tem and Trm cells are functionally heterogeneous and include highly activated as well as more dysfunctional CD8^+^ types.

### Decidual CD8^+^ Teff, Tem, and Trm clusters have distinct cytotoxicity and polyfunctionality.

**Figure 7:**
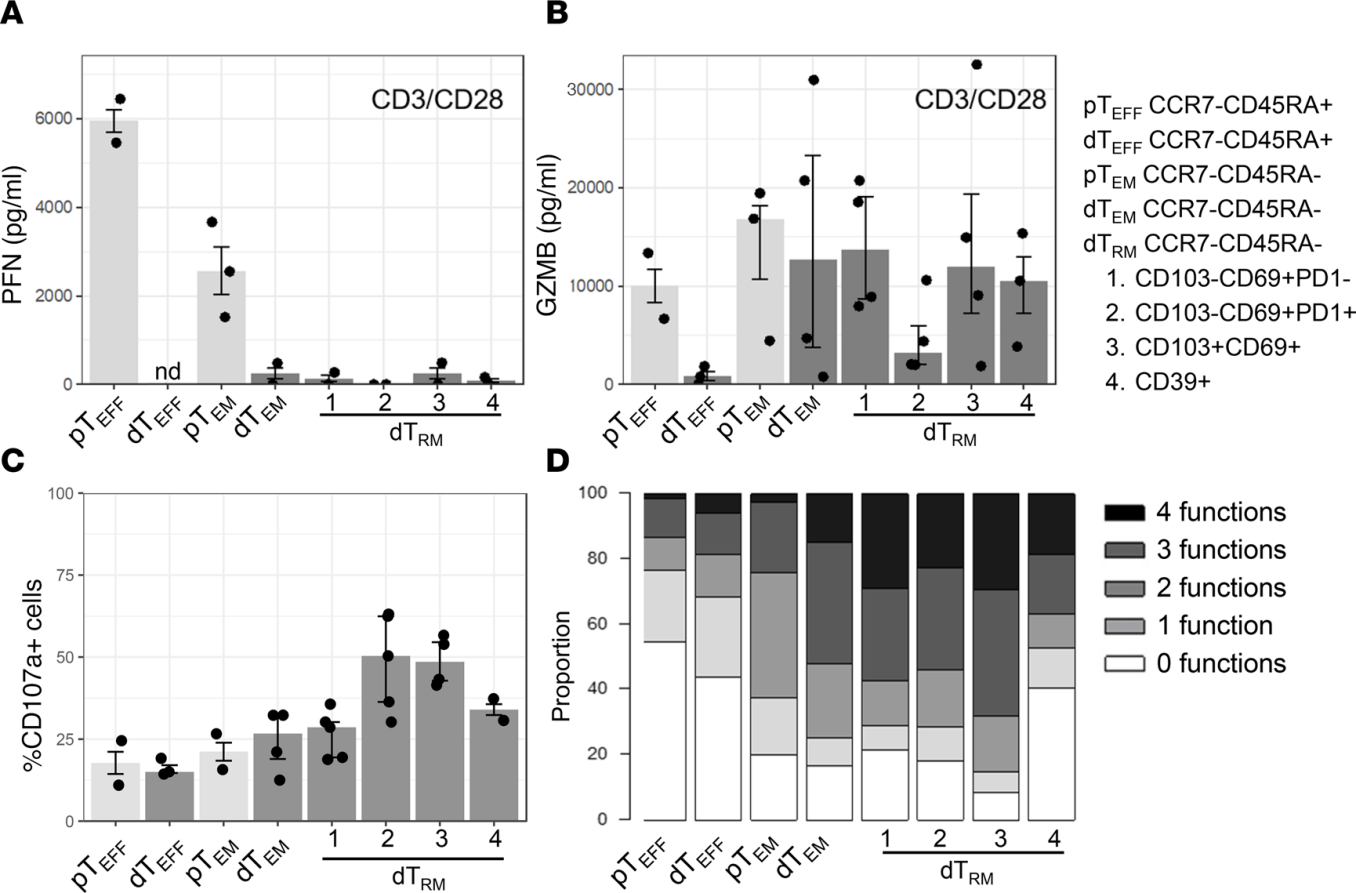
Decidual CD8^+^ Tem clusters have distinct levels of cytotoxicity and polyfunctionality. Graphs depict the concentration of (**A**) perforin (PFN) and (**B**) granzyme B (GZMB) in CD8^+^ T cell culture supernatants upon CD3/CD28 (20 hours) or PMA/I (6 hours) stimulation, as determined by Isoplexis human adaptive immune secretome chip analysis. ND, not done. (**C**) Graphs depict percentage of CD8^+^ T cells expressing CD107a upon PMA/I stimulation for 6 hours as detected by flow cytometry. In **A**–**C**, Bars depict median and interquartile range of *n* = 2–5 samples. (**D**) Graphs depict the proportion of CD8^+^ T cells with 0, 1, 2, 3, and 4 functions. Median of *n* = 2–5 donors is shown.

CD3/CD28 stimulation significantly increased PFN secretion in pTeff and pTem CD8^+^ T cells, whereas none of the decidual CD8^+^ T cell types secreted PFN (Figure 7A), confirming the flow cytometry data (Figure 3A) and previous studies that PFN is absent from decidual CD8^+^ T cell function (11, 12). In contrast, GZMB was secreted by both pTem and dTem and dTrm clusters, but dTeff cells did not secrete GZMB, whereas pTeff cells did (Figure 7B). The GZMB secretion levels by dTem and dTrm types did not have a clear correlation with the expression levels measured (Figure 3C), possibly due to the striking donor-to-donor variation and limited sample size used for the functional analysis. We further assessed the cytolytic capacity of blood and decidual CD8^+^ T cell clusters by assessing their capacity to degranulate (Figure 7C). There was no difference in degranulation, as measured by CD107a^+^ cells upon PMA/I simulation between pTeff and dTeff or between pTem and dTem cells, showing they are functionally similar. However, dTrm cells had equal (Trm1 and Trm4) or higher (Trm2 and Trm3) capacity to degranulate (Figure 7C). We next assessed the polyfunctionality of blood and decidual CD8^+^ T clusters by assessing their capacity to degranulate and produce IFN-γ, TNF-α, and IL-2 simultaneously. First, the frequency of CD107a-, IFN-γ–, TNF-α–, and IL-2–expressing cells was determined (Supplemental Figure 11A). Next, CD8^+^ T cell types were scored for the number of functions they displayed to assess their polyfunctionality. Interestingly, dTem, dTrm1, dTrm2, and dTrm3 had the highest level of polyfunctionality (more than 3 functions) (Figure 7D and Supplemental Figure 11, E and F). Notably, the CD39^+^ dTrm4 cluster had the lowest levels of cytokine production and polyfunctionality, consistent with the dysfunctional state observed above (Figure 6) and described for these cells previously (24, 26, 46). Thus, decidual CD8^+^ T cell clusters are phenotypically and functionally heterogeneous, with significant differences in their capacity to degranulate and exert polyfunctionality.

### Decidual CD8^+^ Tem clusters have distinct survival capacity.

**Figure 8:**
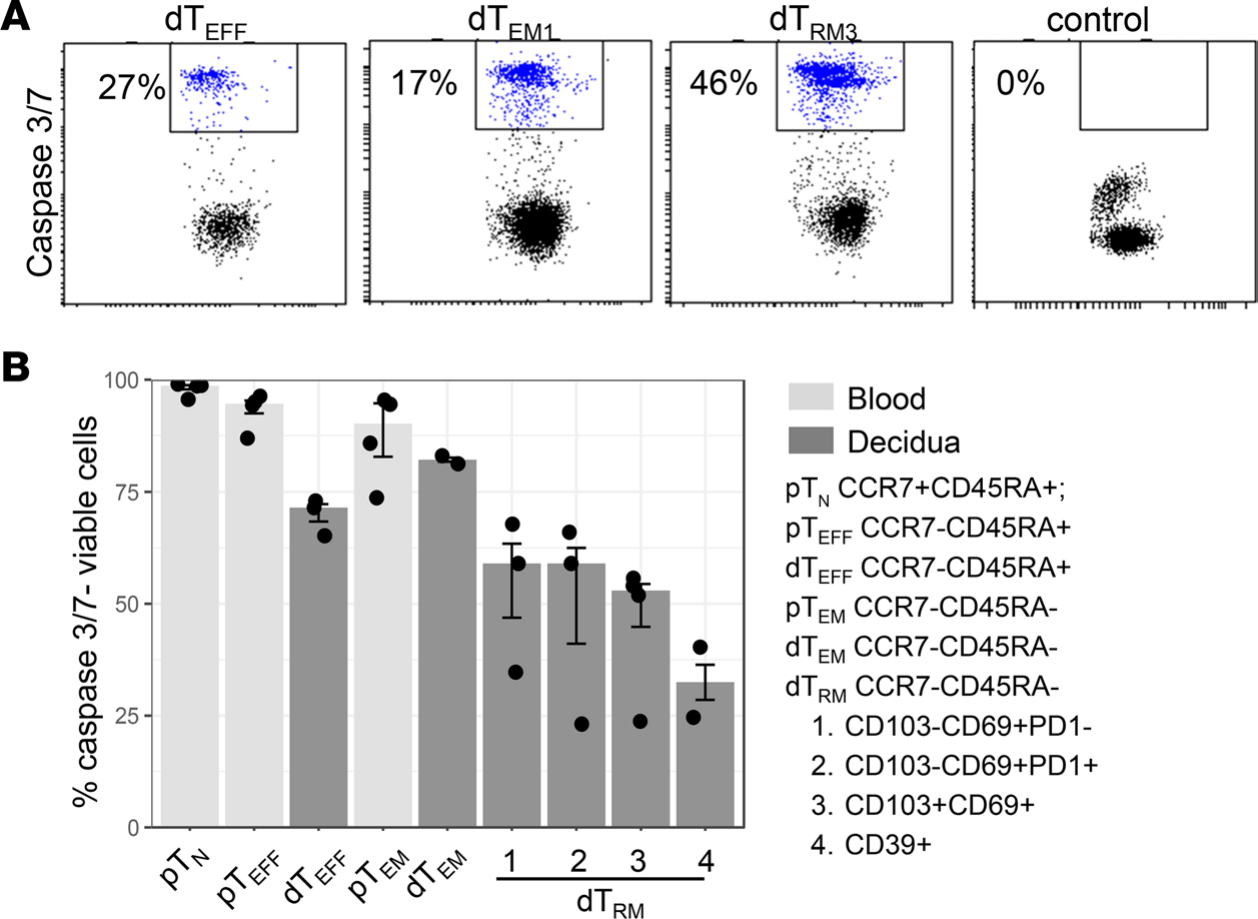
Decidual CD8^+^ Tem clusters have distinct survival capacity. (**A**) Representative dot plots of caspase 3/7 staining in decidual CD8^+^ Teff, Tem1, and Tem4 cells compared with a negative control. (**B**) Graphs depict the percentage of caspase 3/7–negative (–) viable cells in blood and decidual CD8^+^ T cell types. Bars depict median and interquartile range of *n* = 2–4 donors.

The survival capacity of T cells in tissues is essential for their ability to exert their function. Next, blood and decidual CD8^+^ T cell clusters were assessed for their ability to resist apoptosis as a measure of their ability to survive in tissue (47). Blood and decidual CD8^+^ T cell types were sorted as described (Supplemental Figure 1B) and plated in X-Vivo medium without serum and supplements for 20 hours. The frequency of apoptotic cells was determined by a FACS-based caspase-3/7 staining (Figure 8A). All peripheral blood CD8^+^ T cells analyzed (pTn, pTeff, and pTem) had high capacity to resist apoptosis and maintain viability (Figure 8B). Of the decidual CD8^+^ T cell populations tested, dTeff and dTem (CD39^–^CD103^–^CD69^–^PD1^–^) had the highest frequency of viable caspase-3/7^–^ cells, whereas the dTrm populations and in particular the CD39^+^ dTrm4 type had increased frequencies of apoptotic and dysfunctional cells (Figure 8B). Thus, decidual CD8^+^ T cell clusters have significant heterogeneity in their ability to resist apoptosis and survive in tissue.

## Discussion

CD8^+^ memory T cells form the largest fraction of decidual lymphocytes at the maternal-fetal interface in human term pregnancy. We previously reported on their unique features, including their increased expression of activation markers, coinhibitory markers, the low expression of cytolytic molecule PFN, and their ability to degranulate, proliferate, and secrete cytokines upon in vitro stimulation (11, 12). These reports prompted the question whether separate decidual CD8^+^ Tem and Trm types exist that can accommodate maternal-fetal tolerance as well as immunity to infections. Here, we utilized high-dimensional flow cytometry and computational analysis and identified 2 CD8^+^ Tem clusters shared in blood and decidual tissues and 9 CD8^+^ Trm types that are uniquely found in decidua and not in blood. Analysis of their cytokine production demonstrated that pTeff and dTeff as well as pTem and dTem are similar in their cytokine secretion profiles. In contrast, decidual CD8^+^ Trm clusters are heterogeneous, and their cytokine profiles include highly activated as well as more dysfunctional CD8^+^ Trm types. Furthermore, the decreased expression and secretion of PFN, and the increased expression of GNLY by decidual CD8^+^ Teff, Tem, and Trm clusters compared with CD8^+^ Teff and Tem clusters in blood, suggests that decidual CD8^+^ T cell function is dependent on the local tissue factors. Interestingly, the GZMB expression and secretion profiles were highly variable between CD8^+^ T cell clusters, with the dTeff and dTrm2 populations having the lowest secretion of GZMB of all clusters. In addition, the GZMB profiles also had high variability between individual blood and placental donors. This cluster- and donor-dependent variation makes GZMB an interesting functional marker to further investigate in relation to clinical parameters that may influence placental inflammatory states (e.g., mode of delivery, parity, fetal sex, maternal-fetal HLA matching, and HCMV status). Next to their capacity to secrete cytokines and employ cytotoxicity, the capacity for survival in tissues is essential for T cells to exert their function. A limited ability to survive even of highly cytotoxic cells would limit their ultimate response. The apoptosis assay used was shown to be an accurate reflection of the ability of T cells to survive in vivo (47–49), and shows that dTem and dTeff have greater survival capacity ex vivo than dTrm, which include more dysfunctional CD8^+^ T cell types. While these data demonstrate that decidual CD8^+^ T cell clusters have unique phenotypic and functional heterogeneity, the key question is how this heterogeneity relates to their specificity for fetal or viral antigens. Analysis of one donor with positive staining for virus-specific (A2/HCMV^+^) cells in blood and decidual tissues showed that about half of the A2/HCMV^+^ cells in decidua basalis and decidua parietalis overlapped with maternal blood A2/HCMV^+^-specific cells, including their high expression of PFN and GZMB and low expression of inhibitory molecules PD1 and CD39. Decidual virus–specific (A2/HCMV^+^) cells contained an additional CD8^+^ Trm population that was not present in blood, and which had low expression of PFN and GZMB and increased expression of PD1 and CD69. These data suggest that the decidual environment influences the differentiation and cytolytic state of antigen-specific CD8^+^ Trm cells. However, CD8^+^ Tem cells may infiltrate decidual tissues upon infection or inflammation. Interestingly, neither decidual CD8^+^ Tem nor Trm secreted PFN upon CD3/CD28 stimulation, suggesting that mechanisms are in place to control cytotoxicity of dTem and dTrm populations, possibly to prevent allogenic CD8^+^ T cell responses to fetal and placenta antigens. These may also suppress antiviral responses and contribute to the increased pathogenesis of viral infection during pregnancy. Further studies with expanded sample sizes and a further focus on the TCR restriction and clonal expansion of blood and decidual T cells of similar antigen specificity can teach us whether the differences in dysfunctional and cytolytic profiles are due to a restricted influx of CD8^+^ T clones, clonal segregation, or tissue-specific adaptation of matching clones in blood and decidua (50).

Direct comparison of fetus-specific (A2/HY) and virus-specific (A2/HCMV) decidual CD8^+^ T cells, surprisingly, did not identify any differences in their expression of inhibitory, activation, and cytolytic molecules. Both fetus-specific (A2/HY) and virus-specific (A2/HCMV) decidual CD8^+^ T cells had increased frequencies of CD39^+^ and PFN^+^ cells compared with dextramer-negative populations. This suggests that fetus- and virus-specific CD8^+^ T cells are not differentially regulated in decidual tissue and may have a variety of phenotypes and possible functions dependent on tissue type (blood or decidua) and donor. The increased levels of PFN in both fetus- and virus-specific CD8^+^ T cells also suggests that not all fetus-specific CD8^+^ T cell responses are suppressed at the maternal-fetal interface. From here it is also interesting to speculate whether the increase in CD39 on virus-specific T cells results in decreased control of HCMV reactivation or placental infection. This is an important question for further studies investigating larger groups of donors and additional HLA/viral epitopes. While CD39 identified the most dysfunctional CD8^+^ Trm types, CD39 is also an enzyme that, together with CD73, converts adenosine triphosphate into adenosine diphosphate and releases an immunosuppressive form of adenosine in the microenvironment (51). However, microarray gene expression did not detect CD73 expression on freshly isolated or CD3/CD28-stimulated decidual CD8^+^ T cells (12). Other cell types, including decidual macrophages, that express CD73 may work in conjunction with decidual T cells in this pathway of local immune suppression (52).

While previous studies identified many differences in the immune cell types between decidua basalis and decidua parietalis, including increased Treg frequencies in decidua parietalis and distinct dNK types (9, 41, 42, 53), no differences in the CD8^+^ T cell types were found between the 2 tissue sites, confirming previous observations (11). This may suggest that CD8^+^ T cells have similar roles at these 2 sites, whereas Treg and dNK have distinct functions related to the needs for tolerance and trophoblast invasion that are distinct at the site of implantation (decidua basalis) that has deep trophoblast invasion, as opposed to the placental membranes that have more limited trophoblast invasion (53). Similarly, in our study we did not observe clear differences in CD8^+^ T cell populations present in healthy maternal peripheral blood samples drawn just before delivery and peripheral blood samples of healthy nonpregnant donors. Thus, although pregnancy was shown to influence systemic immune populations, including monocyte and Treg function, the peripheral CD8^+^ T cell populations seem less affected.

It is also important to question which CD8^+^ Tem and Trm populations are specific for the pregnant decidua and what CD8^+^ Tem and Trm types may also be found in uterine tissues before pregnancy as well as in other mucosal sites. Similar to decidual CD8^+^ T cells, in endometrial tissues CD8^+^ memory T cell types with low expression of PFN and GZMs have been described (54, 55). Cytotoxicity of endometrial CD8^+^ T cells was lower than cervical CD8^+^ T cells within the same donor, and only endometrial but not cervical CD8^+^ T cell function was regulated by TGF-β (54). This study also demonstrated that endometrial CD8^+^CD103^+^ T cells had reduced cytotoxicity compared with CD8^+^CD103^–^ T cells, suggesting the presence of phenotypically and functionally diverse T cell populations in endometrial tissues. However, how this diversity compares to decidual CD8^+^ memory T cells remains to be determined. The observation that endometrial CD8^+^ T cell cytotoxic activity was dependent on the menstrual cycle and was significantly increased in postmenopausal women compared with premenopausal women suggests that CD8^+^ T cell cytotoxicity is tightly regulated and dependent on uterine receptivity for implantation and pregnancy (54). In humans, tissue-resident CCR7^–^CD45RA^+^ Teff and Trm populations were also shown to be increased in healthy mucosal tissues compared with blood and their proportions were shown to change, depending on donor age (56). In intestinal tissues, CCR7^–^CD45RA^–^ Trm were a dominant CD8^+^ T cell population, whereas in the lung, CCR7^–^CD45RA^+^ Teff were more dominant (56, 57). This may indicate unique tissue type–dependent mechanisms that guide CD8^+^ T cell influx and differentiation to accommodate tissue type–specific requirements for immunity to infection and tolerance for self-, fetus-, microbiome-, and food-derived antigens.

The combination of high-dimensional flow cytometry, computational analysis, and functional testing of human decidual CD8^+^ T cell clusters and the provided association with antigen-specific T cell types is a powerful strategy to translate CD8^+^ T cell phenotype to function to antigen specificity. In contrast to single-cell RNA-seq technologies that have the potential to detect whole genome expression profiles, high-dimensional spectral flow cytometry includes only a limited number of predefined markers. The markers used here were carefully selected based on published gene expression data sets showing enrichment of these activation, coinhibitory, and functional markers in decidual and blood CD8^+^ T cell populations (11–14). Moreover, previous studies demonstrated that for important functional markers, including PFN and GZMB, the RNA expression of decidual CD8^+^ T cells does not correlate with protein expression, highlighting the importance of detecting protein (11, 12). The benefits of detecting protein expression profiles at relatively low cost will also allow for investigation of how clinical data (mode of delivery, parity, fetal sex, maternal-fetal HLA matching, and HCMV status) and placental inflammatory state (e.g., chronic villitis and chorioamnionitis) influence CD8^+^ T cell frequency and function in future studies. Limitations in decidual CD8^+^ T cell yields, low cluster frequencies, and cell number required for functional analysis resulted in some loss of granularity in the functional assessment of CD8^+^ T cell clusters. Further technical developments that can lower cell number requirements (e.g., single-cell secretome assays), increase FACS sorting efficiency, and improve computational clustering tools will improve the translation from CD8^+^ T cell phenotype to function to specificity.

Specificity of CD8^+^ T cells for fetal antigens in humans is difficult to study due to the extremely high polymorphism of the HLA locus and the limited knowledge of allogeneic residues that are expressed by invasive fetal extravillous trophoblasts (EVTs). Maternal allogeneic responses to HLA-C, the only polymorphic molecule expressed by fetal EVTs, are of interest to study but the lack of reagents to study HLA-C–restricted T cells prevents much of this work (1). Moreover, during healthy pregnancies no evidence of antigen-specific immune recognition and direct killing of fetal trophoblasts cells by CD8^+^ T cells has been detected so far (12, 58). Interestingly, 2 studies have highlighted the clonal expansion of placental CD8^+^ T cell populations in infectious and noninfectious villitis, preeclampsia, and miscarriages (4, 59). The next challenge is to relate the clonally expanded placental CD8^+^ T cell populations to antigen specificity and function. These studies are essential for understanding the etiology of chronic chorioamnionitis and villitis characterized by infiltrating maternal CD8^+^ T cell populations. It will provide a basis for the future design of therapeutic strategies aimed to resolve placental inflammation, for example by suppressing excessive antifetal responses or by activating maternal effector cells to clear infection.

## Methods

Discarded placental tissues (gestational age >37 weeks) were obtained from healthy women after uncomplicated pregnancy at term delivered by elective cesarean section or uncomplicated spontaneous vaginal delivery at a local hospital in Cincinnati. All tissues were visually inspected for signs of excessive inflammation (including discoloration, large infarctions, and foul odor) and only healthy tissues were used for further processing. All human tissue used for this research was deidentified, discarded clinical material and no clinical information was available for analysis. The procedures to isolate decidual and peripheral blood CD8^+^ T cells were recently described (12, 60). In short, decidua parietalis from term pregnancy was collected by removing the amnion and delicately scraping the decidua parietalis from the chorion. Decidua basalis was macroscopically dissected from the maternal side of the placenta. Collected decidual tissues were washed, minced, and digested with 0.1% collagenase type IV and 0.01% DNase I (Sigma-Aldrich) for 75 minutes at 37°C in a gently shaking water bath. After digestion, cells were washed and filtered through 100-, 70-, and 40-μm cell strainers (BD, Labware). Lymphocytes were dissolved in 20 mL 1.023 g/mL Percoll (GE Healthcare) and layered on a Percoll gradient consisting of 10 mL 1.080 g/mL and 15 mL 1.053 g/mL Percoll. After density gradient centrifugation (30 minutes, 800*g*), lymphocytes were isolated from the 1.080–1.053 g/mL interface and washed once. Peripheral CD8^+^ T cells were isolated using RosetteSep (StemCell Technologies) followed by Ficoll (GE Healthcare) gradient centrifugation (20 minutes, 800*g*). All blood and lymphocyte preparations were washed once and directly stained for FACS on a BD FACSAria II (Supplemental Figure 1) or for analysis on a Cytek Aurora spectral flow cytometer.

### Flow cytometry.

Antibodies and isotype controls used for flow cytometric analysis and FACS are listed in Supplemental Table 3. For surface staining, cells were stained for 30 minutes in RPMI containing penicillin/streptomycin and 10% newborn calf serum. For intracellular staining, cells were fixed and permeabilized using the CytoFix/CytoPerm kit (BD). Analysis was performed on a Cytek Aurora or BD Fortessa. FACS was done using a BD FACSAria II. All flow cytometric data were acquired using equipment maintained by the Research Flow Cytometry Core in the Division of Rheumatology at Cincinnati Children’s Hospital Medical Center.

### Dextramer staining and flow cytometric analysis.

Decidual and blood samples were stained for CD45, CD14, CD56, and CD4. The CD45^+^CD14^–^CD56^–^CD4^–^ cells were selected and purified using a BD FACSAria II. CD8 was not included in the sort panel as it has been shown to block dextramer binding. After sorting, the CD45^+^CD14^–^CD56^–^CD4^–^ cells were stained with HLA*02:01_HCMV_ NLVPMVATV and HLA-A*02:01_HY_FIDSYICQV dextramers (Immudex) at room temperature for 10 minutes followed by staining for cell-surface markers, including CD8. Data acquisition was performed on a Cytek Aurora and data were analyzed using FlowJo software (BD Biosciences).

### Data processing and computational analysis.

Initial data processing and gating on CD45^+^CD14^–^CD3^+^CD56^–^CD4^–^CD8^+^ T cells was performed using FlowJo software. FCS files of CD45^+^CD14^–^CD3^+^CD56^–^CD4^–^CD8^+^ T cells were exported for further analysis in R software version 4.1.0 (https://cran.r-project.org/bin/macosx/base/). In R, values of the remaining markers for the analysis were arcsinh transformed with a cofactor of 6000 (61). To explore the similarity of samples and to check for outliers and batch effects, MDS plots based on median marker expressions for all samples, including technical controls, were generated as previously described (62). Technical control samples were not included in any further analyses. A PCA-based NRS analysis was used to rank markers by their ability to explain the variance observed in the samples (39, 62). To ensure each sample and tissue contribute equally, unsupervised clustering was performed on 50,000 randomly selected cells in each sample. Expression of CCR7, CD45RA, CD28, CD27, CD39, CD103, CD69, PD1, and ICOS was included in the FlowSOM algorithm (R package FlowSOM, version 2.0.0) with 15 predefined clusters in the meta-clustering step (40). Scaled median expression values per marker per cluster were visualized in a heatmap using R package pheatmap (version 1.0.12) as previously described (62). Clusters 1 and 3 had only minor differences in expression patterns and were manually merged into cluster 1 (Supplemental Figure 4). Clusters were further visualized in density plots of fluorescence intensities and by overlaying them after the nonlinear dimensionality reduction technique, uniform manifold approximation and projection (UMAP), was applied to the cell-surface markers of randomly selected 300,000 cells (20,000 cells per file) (R package UMAP, version 0.2.8.0) (63). UMAPs were plotted including cells from all sample types, and stratified for blood, decidua basalis, and decidua parietalis while including 2D density contour lines based on cells from all samples. For each cluster, cell frequencies between decidua basalis and decidua parietalis as well as in blood and decidua (basalis and parietalis combined) were compared using Wilcoxon’s signed-rank test or Wilcoxon’s rank-sum test, respectively (compare means function in R package ggpubr, version 0.4.0). Median expression of functional markers (PFN, GNLY, GZMB) in each cluster was calculated for all samples that had at least 100 cells in the respective cluster. Median expression values were compared between blood and decidual samples using Wilcoxon’s rank-sum test. GNLY median expression values of decidua samples were compared between all clusters using the Kruskal-Wallis test. *P* values for the comparisons of cell frequencies and median expressions between blood and decidua were false discovery rate (FDR) adjusted.

### T cell survival assays.

Blood and decidual CD8^+^ Tem cell populations (sorted as shown in Supplemental Figure 1, B and C) were plated directly after FACS at a density of 2.5 × 10^4^ cells per well in a round-bottom 96-well plate in 100 μL X-Vivo 10, human source medium (Lonza) without supplements. After 20 hours, an Alexa Flour 488–labeled caspase-3/7 detection antibody (Thermo Fisher Scientific) was added for 10 minutes to identify apoptotic cells. Thereafter, cells were directly analyzed on a BD Fortessa flow cytometer.

### Cytokine secretion assays.

Blood and decidual CD8^+^ Tem cell populations (sorted as shown in Supplemental Figure 1, B and C) were plated directly after FACS at a density of 2.5 × 10^4^ cells per well in a round-bottom 96-well plate in 25 μL X-Vivo medium supplemented with 5% human serum and 50 U/mL IL-2. CD8^+^ T cell populations were stimulated using either anti-CD3/anti-CD28 beads (1 μg/mL) for 20 hours or phorbol myristate acetate (PMA) and ionomycin (each at 1 μg/mL) for 6 hours. Supernatants were collected, snap frozen, and stored at –80°C. Eleven microliters of undiluted supernatants were analyzed on Isoplexis Human Adaptive Immune CodePlex Secretome chips to detect levels of GM-CSF, GZMB, IFN-γ, IL-2, IL-4, IL-5, IL-6, IL-7, IL-8, IL-9, IL-10, IL-13, IL-15, IL-17A, IP-10, MCP-1, MIP-1α, MIP-1β, PFN, sCD137, TNF-α, and TNF-β.

### Degranulation and polyfunctionality assays.

Blood and decidual CD8^+^ Tem cell populations (sorted as shown in Supplemental Figure 1, B and C) were plated directly after FACS at a density of 7.5 × 10^4^ cells per well in a round-bottom 96-well plate in 25 μL X-Vivo medium supplemented with 5% human serum and 50 U/mL IL-2. To assess degranulation and cytokine production, CD8^+^ T cell populations were stimulated using PMA/I each at 1 μg/mL in the presence of monensin (BioLegend) and 250 ng/mL anti-CD107a PerCP-Cy5.5 (BioLegend) for 6 hours. The CD8^+^ T cells were then collected and fixed using 4% paraformaldehyde for 30 minutes. Cells were stained for CD45 and CD8 for 20 minutes on ice, permeabilized using the CytoFix/CytoPerm kit (BD), and stained for IFN-γ, TNF-α, and IL-2 for 20 minutes on ice and directly analyzed on a BD Fortessa flow cytometer.

### Statistics.

All statistical analyses and visualizations were performed using R software (version 4.1.0). Plotting of data was done using the ggplot2 R package (version 3.3.5) unless otherwise stated. In box-and-whisker plots, the medians are shown, and the hinges represent the first and third quartile. The whiskers are the smallest and largest values after exclusion of outliers (greater than the 75th percentile plus 1.5 times the interquartile range [IQR], or less than 25th percentile minus 1.5 times the IQR). In bar graphs, the medians are shown as bar and the IQR as error bars. *P* values and adjusted *P* values of less than 0.05 were considered significant. All statistical tests were performed 2-sided.

### Study approval.

Discarded healthy human placental materials (gestational age >37 weeks) were collected at a local hospital. All human tissue used for this research was deidentified, discarded clinical material and no clinical information was available for analysis. The Institutional Review Board (IRB) determined that this use of placental materials is Not Human Subjects Research.

### Data availability.

Data are available in the Supplemental Data Value XLS file. Flow cytometry data and R code are available upon reasonable request.

## Author contributions

SA and TT designed the research. SM, AA, and ZK performed the research. SM, AA, NS, SA, and TT analyzed data. ED contributed materials. SM, NS, SA, and TT visualized data. SA and TT acquired funding. SM, SA, and TT wrote the original draft of the manuscript, which was reviewed and edited by NP, DAH, CAC, SA, and TT.

## Supplementary Material

Supplemental data

Supporting data values

## Version 1 09/08/2023

Electronic publication
