# Supplementary material for: Antigen-specific decidual CD8+ T cells include distinct effector memory and tissue-resident memory cells
Source: JCI Insight. 2023 Sep 8;8(17):e171806. doi: 10.1172/jci.insight.171806 (PMC10544202; doi:10.1172/jci.insight.171806)
Supplement: Supplemental data [file jciinsight-8-171806-s256.pdf]

SUPPLEMENTARY INFORMATION

ANTIGEN-SPECIFIC DECIDUAL CD8+ T CELLS INCLUDE DISTINCT EFFECTOR MEMORY AND TISSUE RESIDENT MEMORY CELLS

Shweta Mahajan, Aria Alexander Zachary Koenig, Nicolas Saba, Nina Prasanphanic, David A. Hildeman, Claire A. Chougnet, Emily DeFranco, Sandra Andorf and Tamara Tilburgs

List of Supplementary Figures and Tables

Supplementary Figure S1: Gating Strategy

Supplementary Figure S2: Multidimensional Scaling Plots (MDS) separate CD8+ T cells by tissue type and not by batch.

Supplementary Figure S3: Non redundancy score (NRS) are used to select markers for clustering.

Supplementary Figure S4: Cluster 1 and 3 are phenotypically identical

Supplementary Figure S5: Expression profiles of FlowSOM clusters

Supplementary Figure S6: Cell Frequency per cluster are not different between decidua basalis and decidua parietalis nor between non pregnant and pregnant blood CD8+ T cells.

Supplementary Figure S7: Blood and decidual CD8+ T cell clusters have distinct expression of cytolytic granules.

Supplementary Figure S8: Virus-specific CD8+ T cells are distinct in blood and decidua

Supplementary Figure S9: Decidual virus- and fetus-specific CD8+ T cells have similar features of inhibition and cytotoxicity

Supplementary Figure S10: Decidual CD8+ T_{EM} and T_{RM} clusters have distinct cytokine secretion profiles.

Supplementary Figure S11: Decidual CD8+ T_{EM} and T_{RM} clusters have distinct levels of poly-functionality

Supplementary Table S1: 21 Parameter Cytek Aurora panel.

Supplementary Table S2: Sorting of CD8+ T cell clusters for functional testing.

Supplementary Table S3: List of antibodies used.

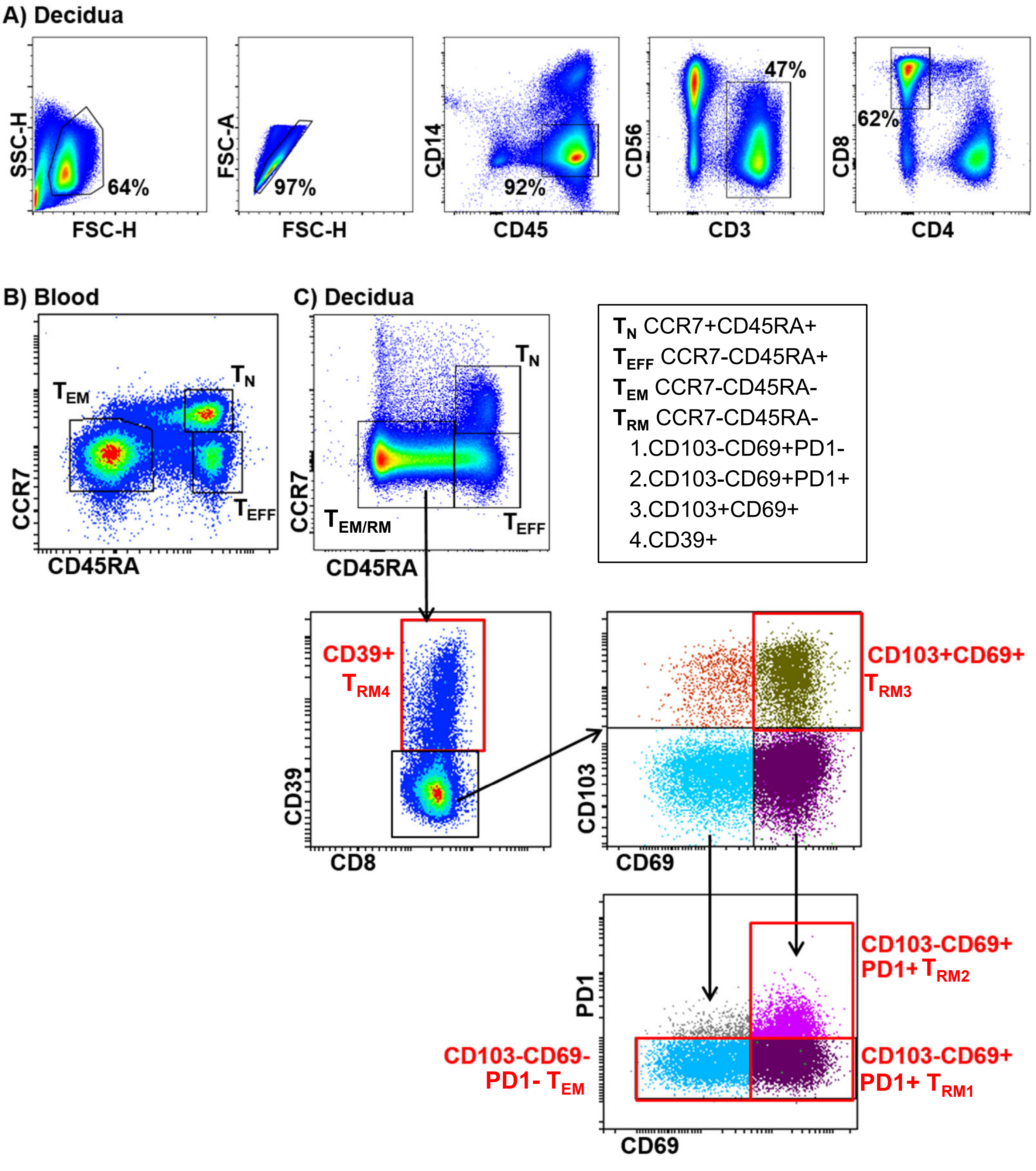

Supplementary Figure S1. Gating Strategy

A) CD8+ T cells were selected by gating on live and single cells. CD45+ CD14- CD56- CD3+ CD4- CD8+ cells were selected in subsequent plots. B) Peripheral blood CD8+ T cells were purified into CD45RA+CCR7+ Naïve (T_N), CD45RA+CCR7+ Effector (T_{EFF}), and CD45RA-CCR7- Effector-Memory (T_{EM}) CD8+ T cells by FACS sort. C) Decidual CD8+ T cells were purified into CD45RA+CCR7+ Naïve (T_N), CD45RA+CCR7+ Effector (T_{EFF}) CD8+ T cells, CD45RA-CCR7- CD8+ Effector-Memory (T_{EM}) and four types of CD45RA-CCR7- Resident-Memory (T_{RM})CD8+ T cells based on the expression of CD39+ (T_{RM4}); CD39-CD103+CD69+ (T_{RM3}); CD39-CD103-CD69+PD1- (T_{EM1}); and CD39-CD103-CD69+PD1+ (T_{EM2}) populations.

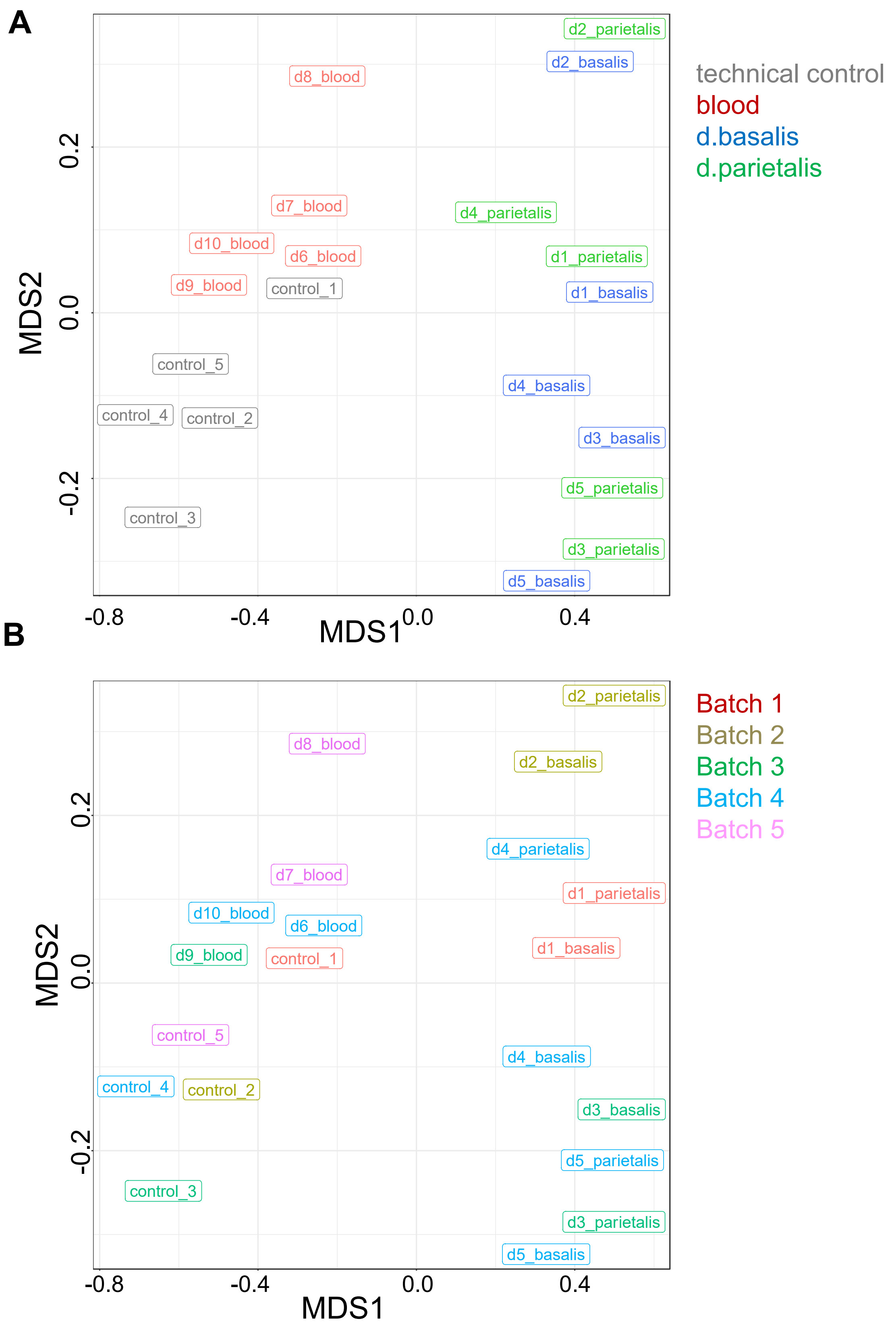

Supplementary Figure S2. Multidimensional Scaling Plots (MDS)

MDS plots separate CD8+ T cells by tissue type (A) and not by batch (B).

A

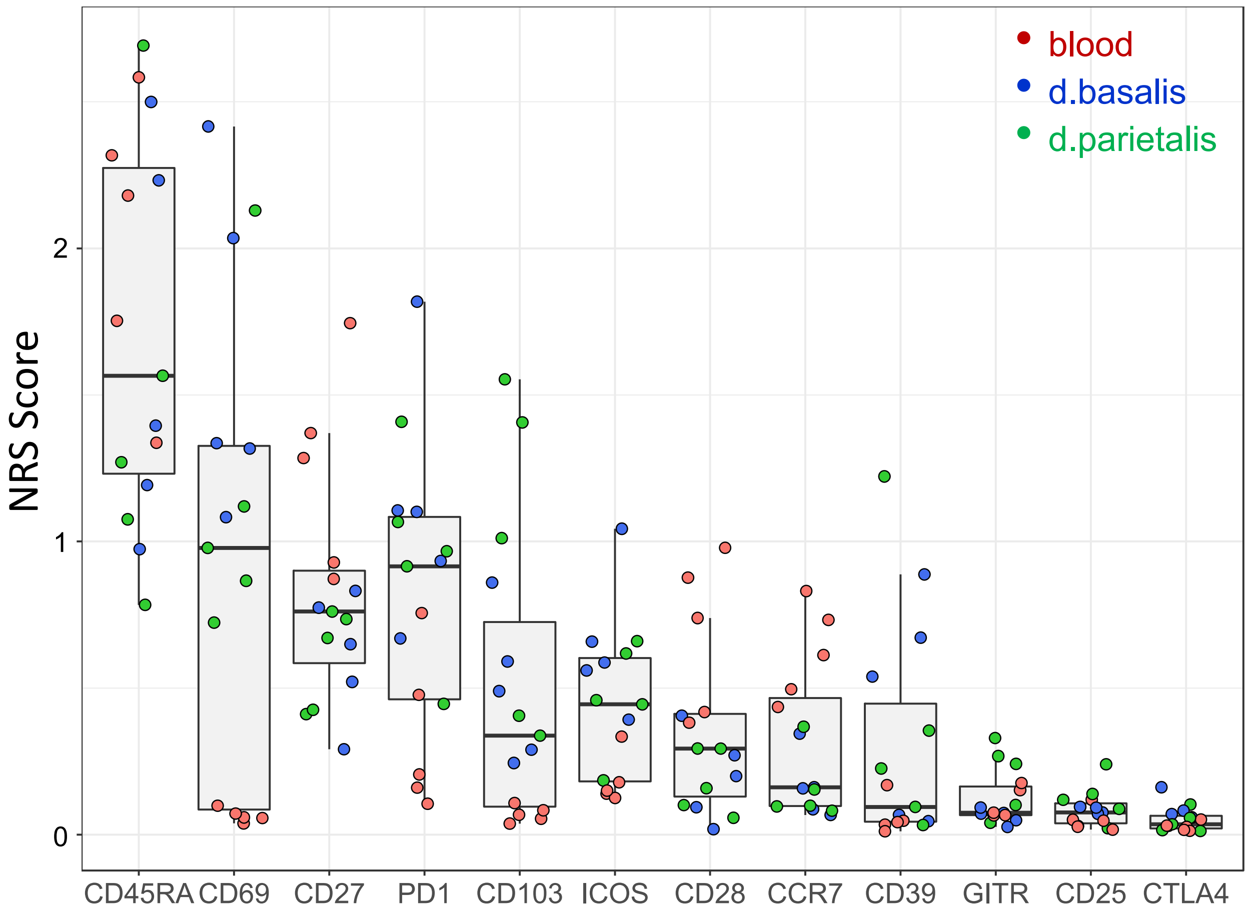

B

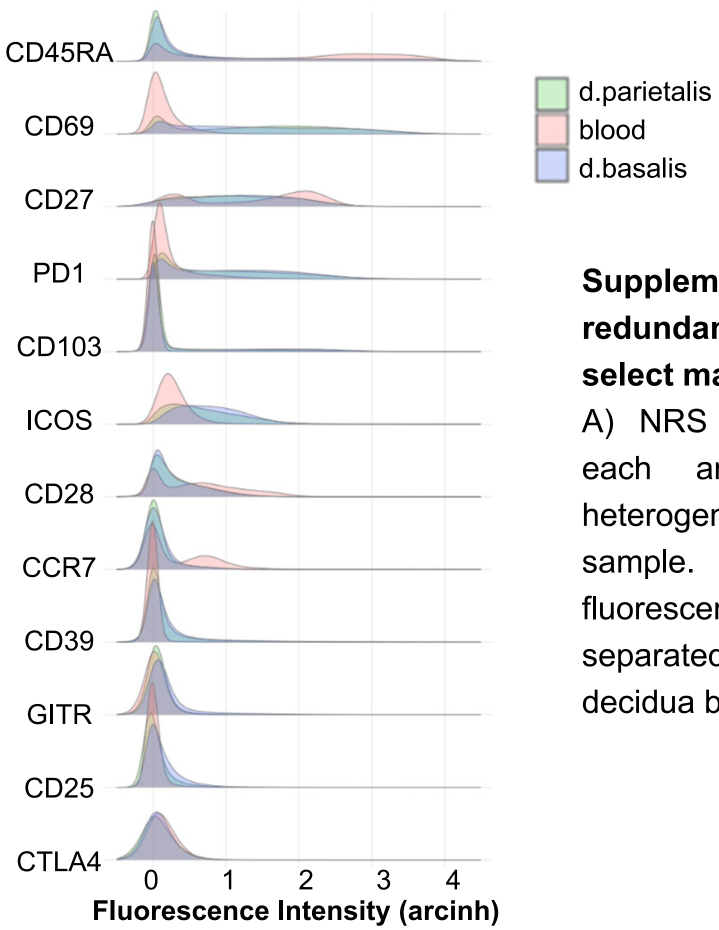

Supplementary Figure S3. Non redundancy score (NRS) are used to select markers for clustering

A) NRS plot depicts contribution of each antigen to CD8+ T cell heterogeneity. Each dot represents one sample. B) Density plots depict fluorescence intensity of each marker separated by tissue type (blood, decidua basalis and decidua parietalis

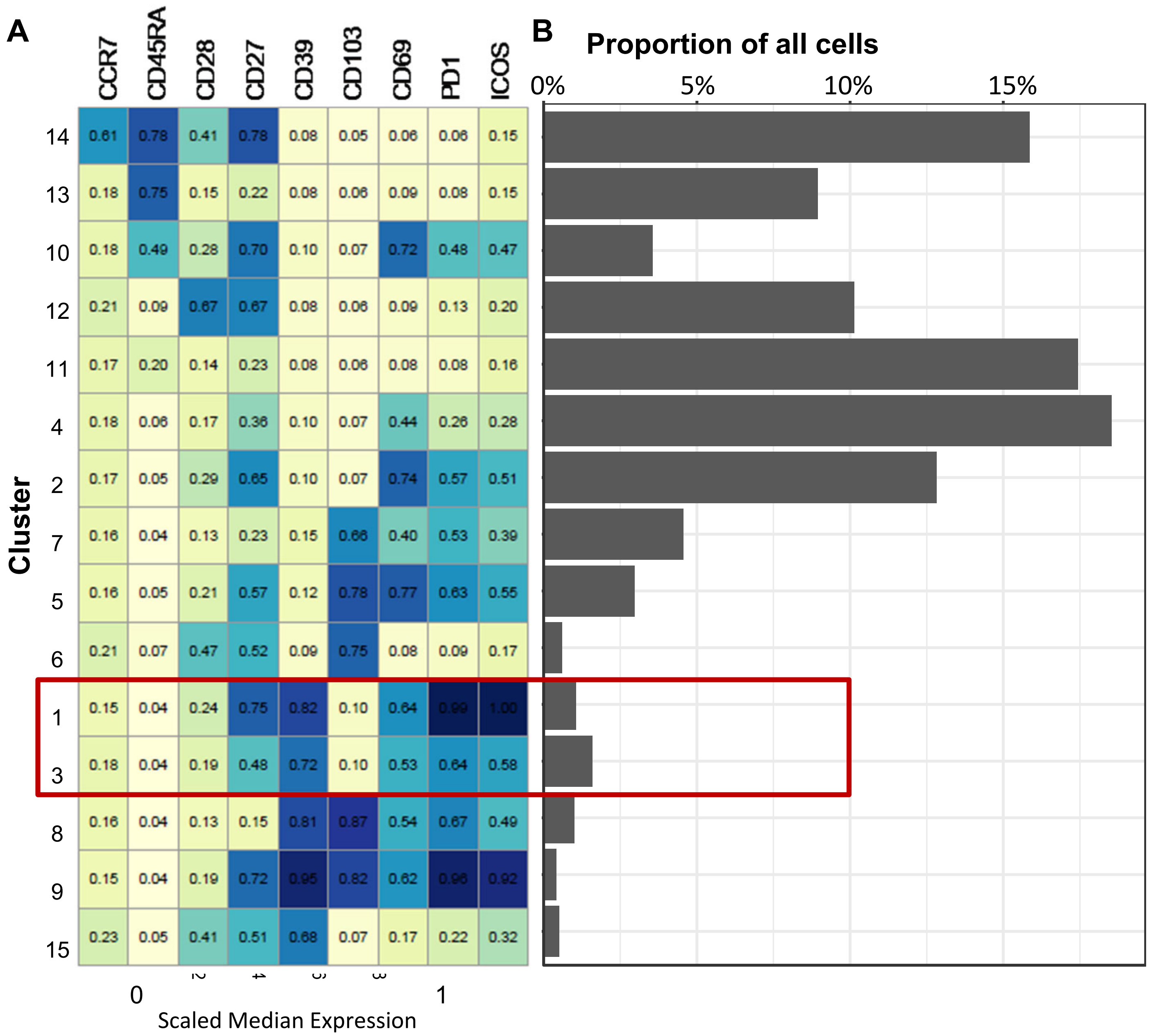

Supplementary Figure S4. Cluster 1 and 3 are phenotypically identical

15 FlowSOM populations were generated based on expression profiles of CD45+CD14-CD56-CD3+CD4-CD8+ T cells from the blood (n=5), decidua basalis (n=5) and decidua parietalis (n=5). A) Heat map shows the arcsinh transformed scaled median fluorescence intensity of the specified markers for the 15 FlowSOM populations. B) Graph depicts the frequency of cells in each cluster as percentage of all analyzed cells. The red box identifies 2 small clusters, cluster 1 and cluster 3 that are phenotypically identical and therefore merged into cluster 1 for further analysis.

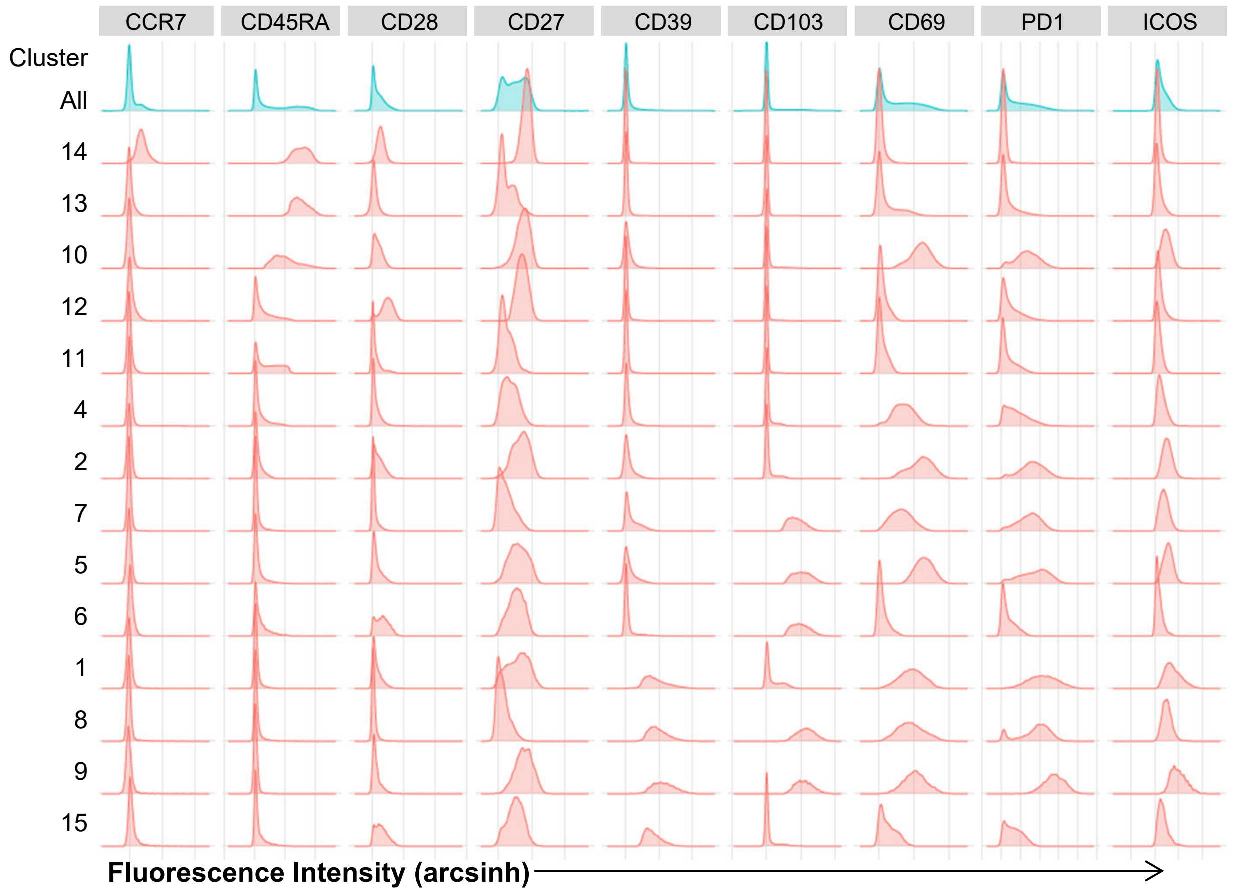

Supplementary Figure S5. Expression profiles of FlowSOM clusters.
Density plots show the arcsinh transformed fluorescence intensity of each marker used for FlowSOM clustering for each of the CD8+ T cell clusters.

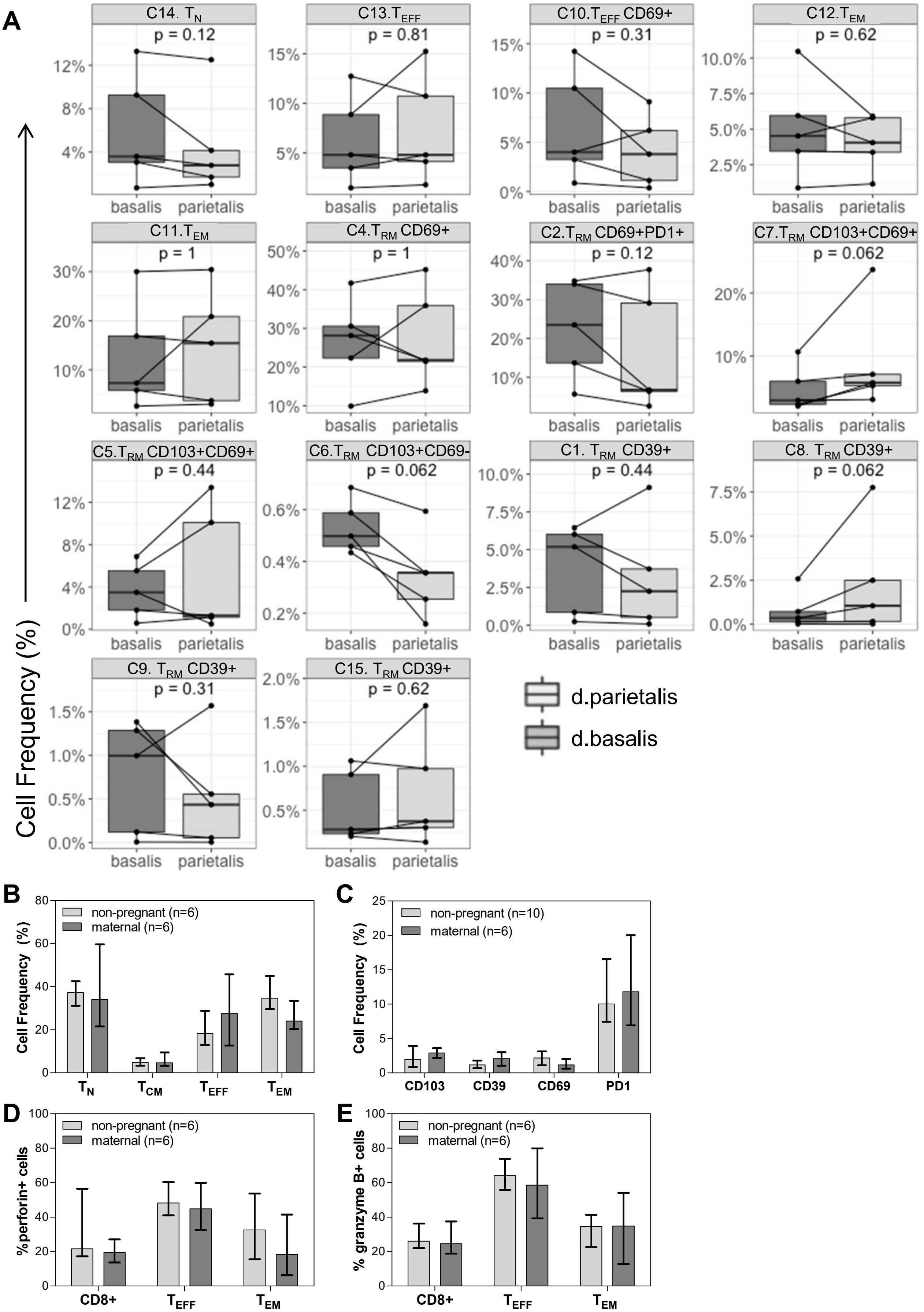

Supplementary Figure S6. Cell Frequency per cluster are not different between decidua basalis and decidua parietalis nor between non pregnant and pregnant blood CD8+ T cells.

A) Graphs depict cell frequencies per cluster in decidua basalis and decidua parietalis. Boxes depict median and interquartile range. P. values determined by Wilcoxon signed-rank test. Graphs depict B) the percentage CD45RA+CCR7+ T_N, CD45RA-CCR7+ T_{CM}, CD45RA+CCR7- T_{EFF}, and CD45RA-CCR7- T_{EM} cells; and C) the percentage CD103, CD39, CD69 and PD1 positive cells in non-pregnant blood and maternal blood CD8+ T cells. The percentage D) perforin and E) granzyme B positive cells are shown in total CD8+ T cells, CD45RA+CCR7- T_{EFF}, and CD45RA-CCR7- T_{EM} CD8+ T cells in non-pregnant and maternal blood. Bars and lines depict median and interquartile range of 6-10 donors.

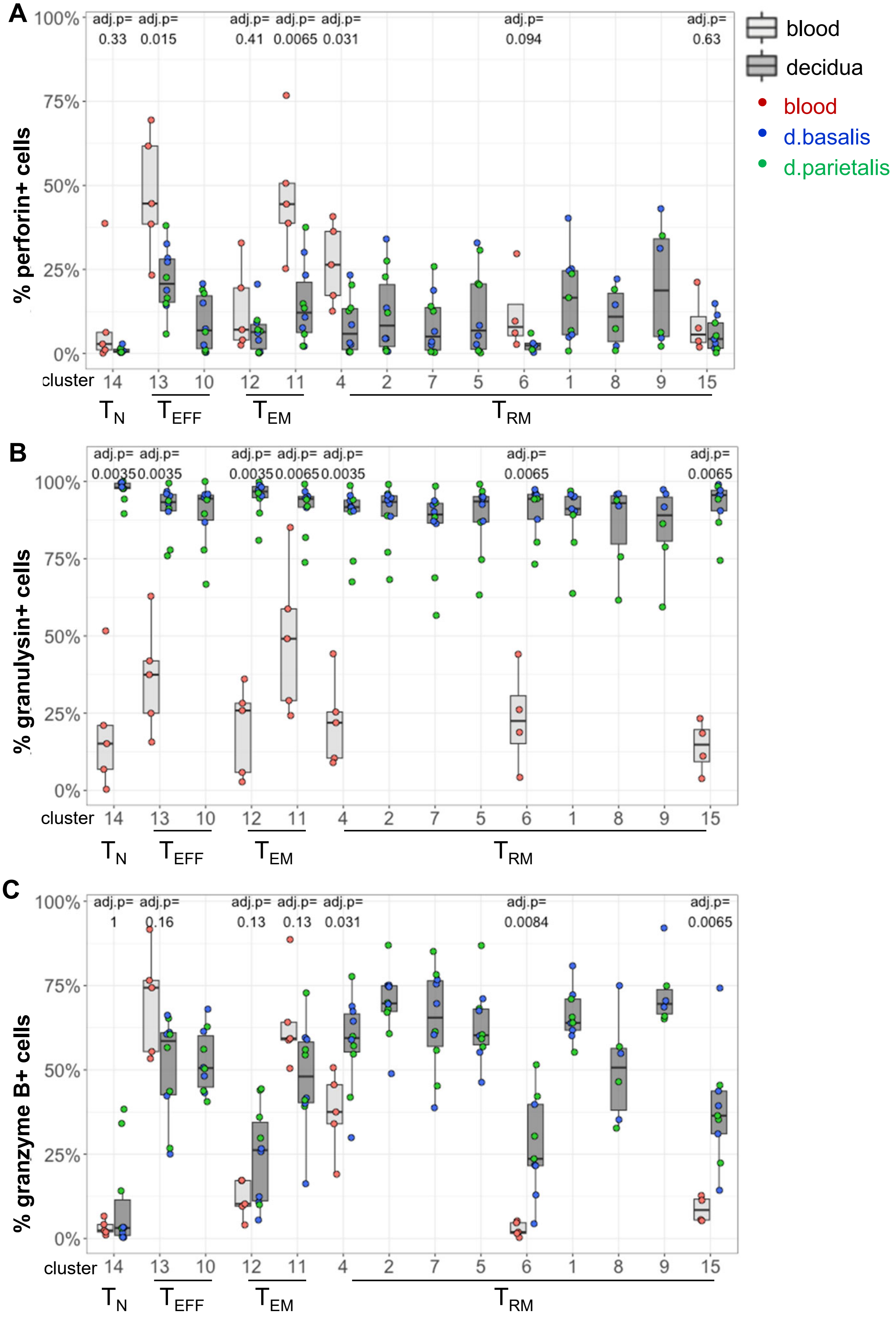

Supplementary Figure S7. Blood and decidual CD8+ T cell clusters have distinct expression of cytolytic granules. Graphs depicts the frequency of A) perforin+, B) granulysin+ and C) granzyme B+ cells in the CD8+ T cell clusters present in blood (n=5) (light grey boxes) and d.basalis (n=5) and d.parietalis (n=5) (dark grey boxes). Boxes depict median and interquartile range. P values by Wilcoxon rank sum test and FDR-adjusted.

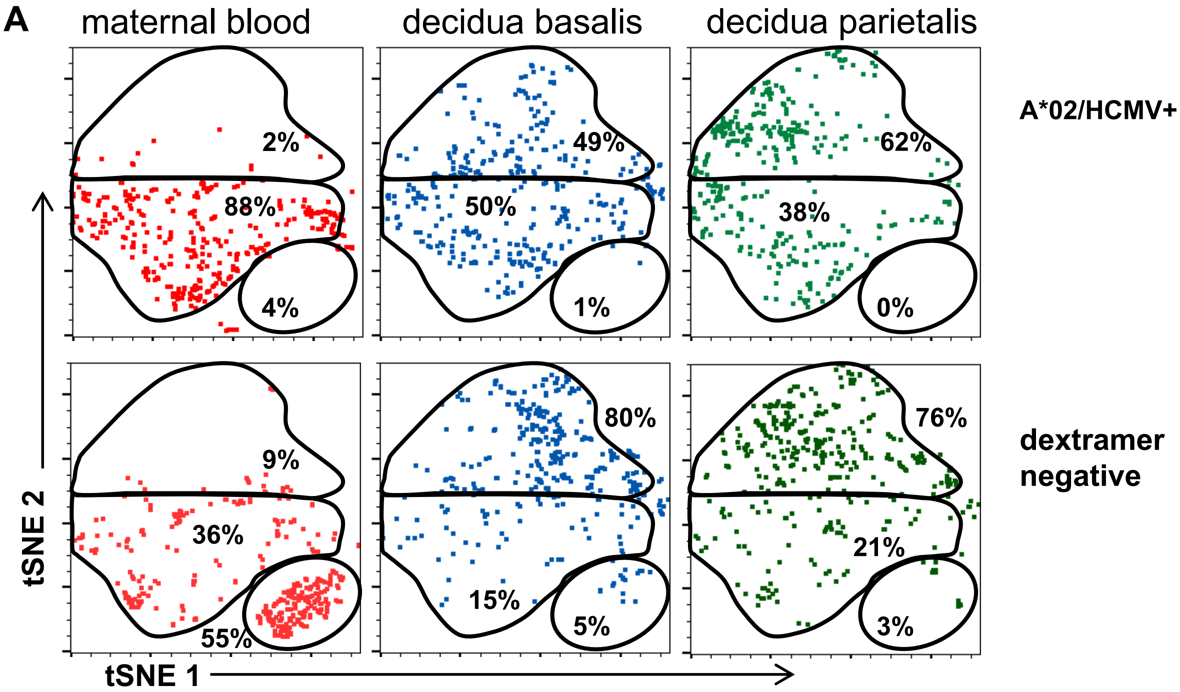

Supplementary Figure S8. Virus-specific CD8+ T cells are distinct in blood and decidua. tSNE plots of HLA-A2/HCMV specific cells (top) and dextramer negative cells (bottom) in maternal blood (red), decidua basalis (blue) and decidua parietalis (green) of one donor.

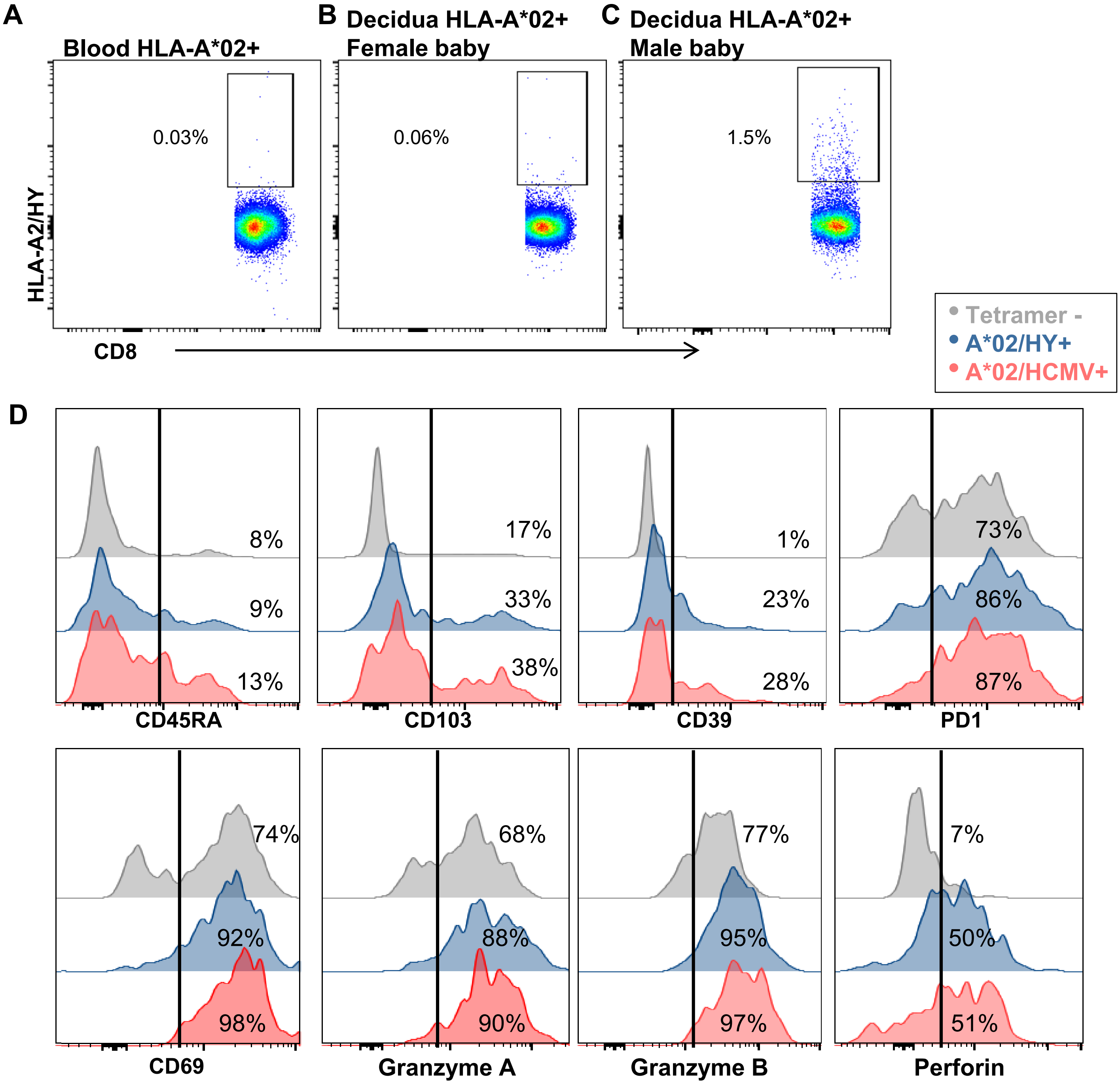

Supplementary Figure S9. Fetus and virus-specific CD8+ T cells have similar phenotypes. Representative FACS plots of HLA-A*02/HY dextramer staining in A) an HLA-A*02+ blood donor without any positive HY population B) an HLA-A*02+ decidua donor after a pregnancy with a female fetus without any positive HY population and C) a HLA-A*02+ decidua donor after a pregnancy with a male fetus with a clear HY population. D) Representative FACS histograms of CD45RA, CD103, CD39, PD1, CD69, granzyme A, granzyme B and perforin expression in dextramer negative (grey), HLA-A2/HY specific (blue) and HLA-A2/HCMV specific (red) decidual CD8+ T cells.

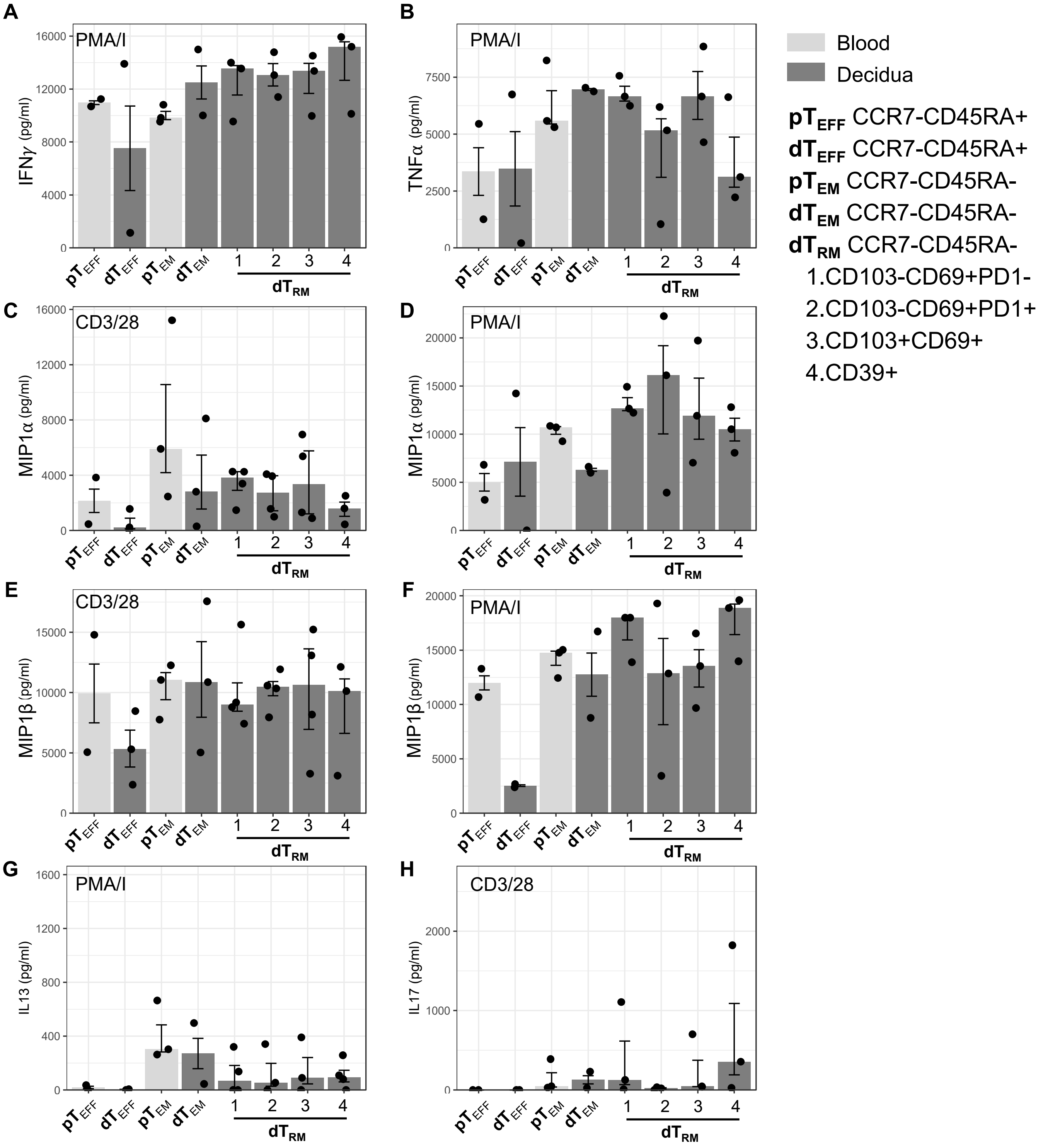

Supplementary Figure S10. Decidual CD8+ T_{EM} and T_{RM} clusters have distinct cytokine secretion profiles. Graphs depict the concentration of A) IFNγ; B) TNFα; C-D) MIP1α; E-F) MIP1β; G) IL13 and H) IL17 in the supernatants upon CD3/28 or PMA/I stimulation (as indicated) detected by the Isoplexis human adaptive immune secretome chips. Bars depict median and interquartile range of 2 – 5 donors.

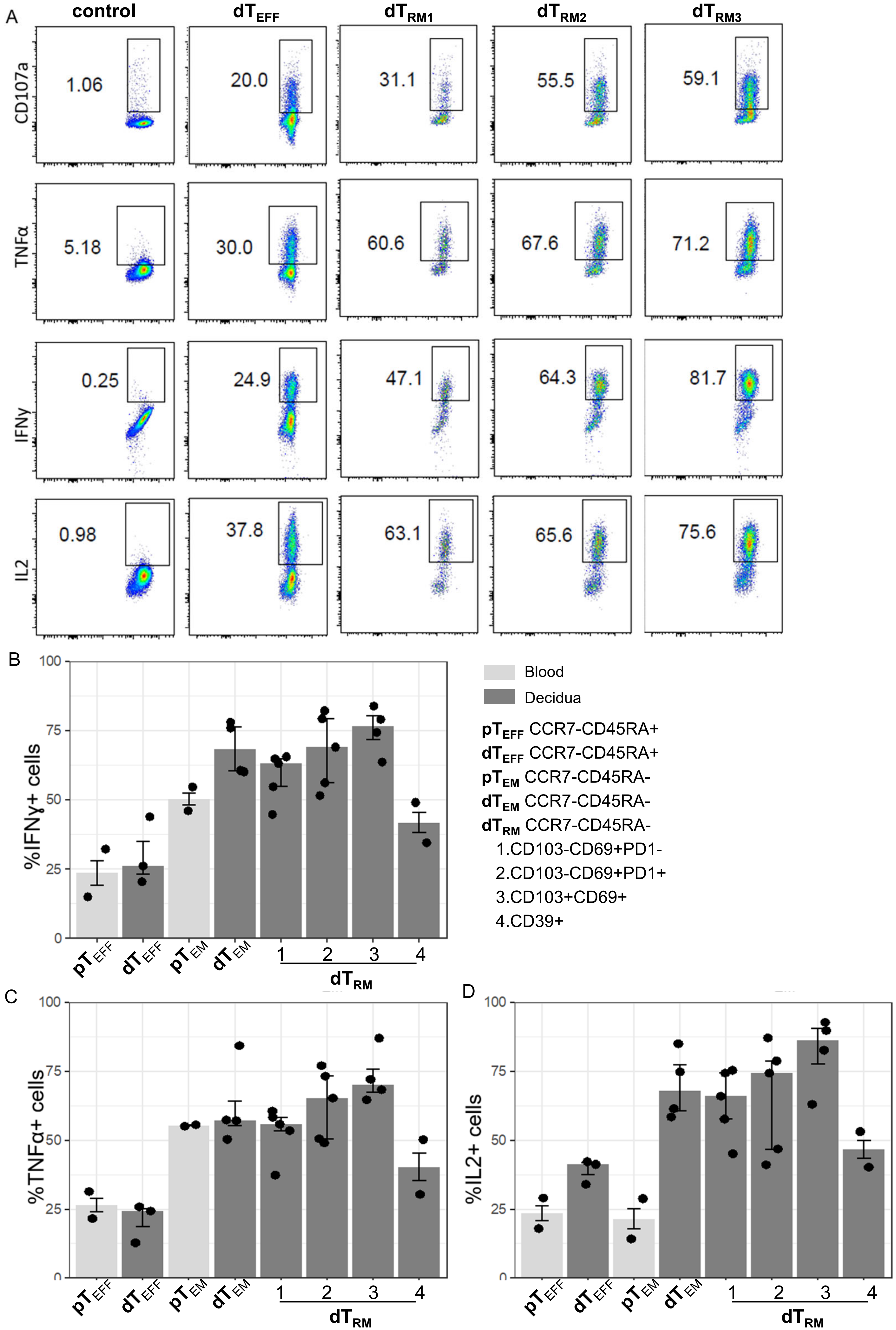
A
control
dT EFF
dT RM1
dT RM2
dT RM3
CD107a
1.06
20.0
31.1
55.5
59.1
TNFα
5.18
30.0
60.6
67.6
71.2
IFNγ
0.25
24.9
47.1
64.3
81.7
IL2
0.98
37.8
63.1
65.6
75.6
B
%IFNγ+ cells
Blood
Decidua
pT EFF CCR7-CD45RA+
dT EFF CCR7-CD45RA+
pT EM CCR7-CD45RA-
dT EM CCR7-CD45RA-
dT RM CCR7-CD45RA-
1.CD103-CD69+PD1-
2.CD103-CD69+PD1+
3.CD103+CD69+
4.CD39+
C
%TNFα+ cells
D
%IL2+ cells
pT EFF
dT EFF
pT EM
dT EM
1
2
3
4
dT RM
0
25
50
75
100

Supplementary Figure S11 continued

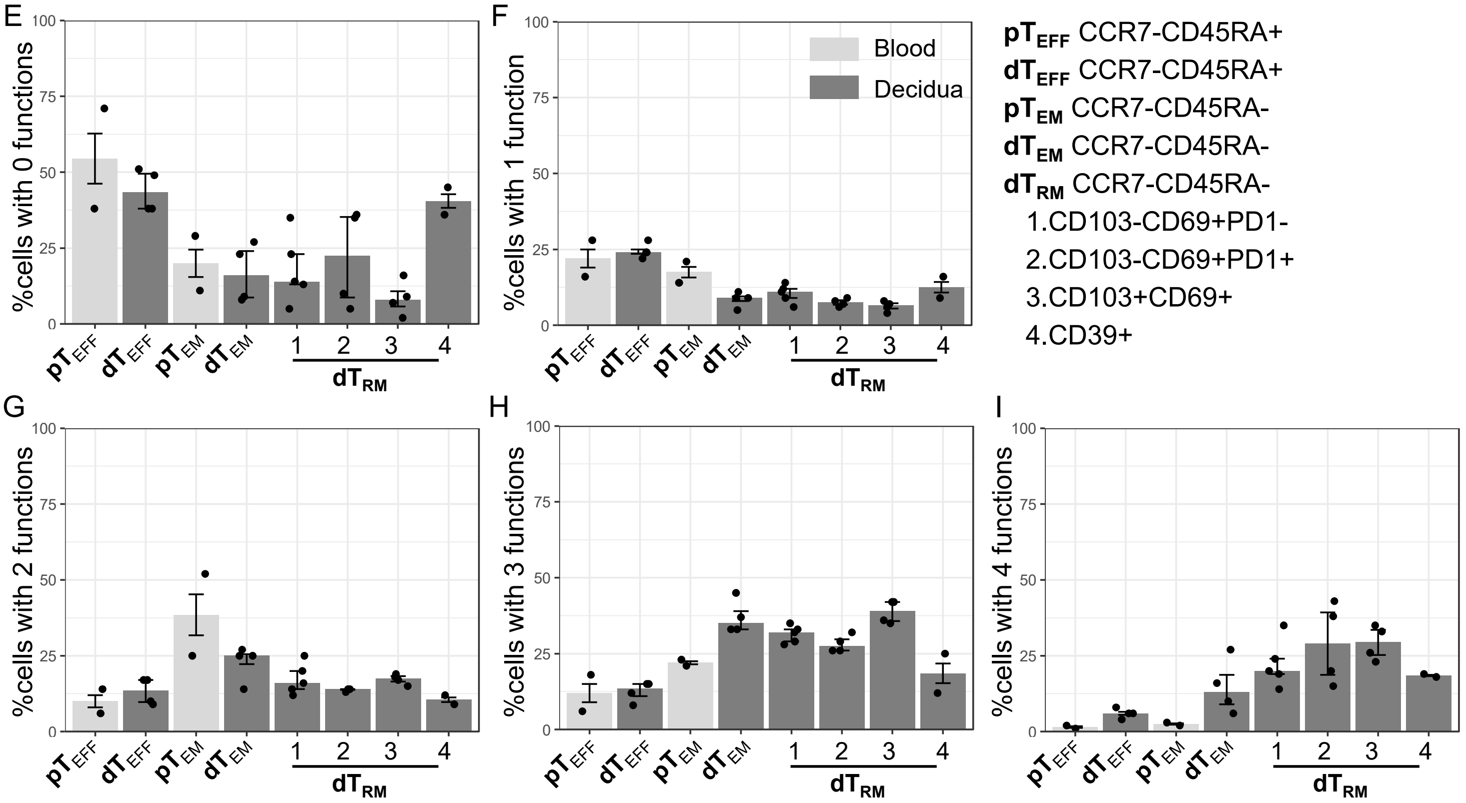

Supplementary Figure S11. Decidual CD8+ T_{EM} and T_{RM} have distinct levels of poly-functionality

A) Representative FACS plots depicting CD107a, IFNγ, TNFα, and IL2 expression in decidual T_{EFF}, T_{RM1}, T_{RM2} and T_{RM3} cells stimulated with PMA/I for 6 hours compared to unstimulated control CD8+ T cells. Graphs depict the percentage of cells within T_{EFF}, T_{EM} and four T_{RM} types positive for B) IFNγ, C) TNFα and D) IL2 as well as the percentage of cells that have E) 0, F) 1, G) 2, H) 3 and I) 4 combined functions (CD107a, IFNγ, TNFα, and IL2). Bars depict median and interquartile range of 2 - 5 donors.

Laser	Peak	Fluor	Antigen
UV	UV2	BUV395	CD4
355	UV7	BUV496	CD56
	UV14	BUV737	CD3
	UV16	BUV805	CD8
VIOLET	V1	BV421	CD103
405	V3	P.Blue	Perforin*
	V7	BV510	CD14
	V10	BV605	CD69
	V11	BV650	CD28
	V13	BV711	ICOS
	V14	BV750	CD27
BLUE	B2	AF488	GNLY*
488	B3	SB550	CD45
YG	YG1	PE	CD25
561	YG3	PE/Dzle594	CD39
	YG5	PE/Cy5	CD45RA
	YG9	PE/Cy7	PD1
RED	R1	APC	GITR
640	R2	AF647	GZMB*
	R4	AF700	CCR7
	R7	APC/Fire750	CTLA4*

Supplementary Table S1. 21 Parameter Cytek Aurora panel.
*Intracellular Staining

	Name	Phenotype	Clusters
Blood	pT_N	CD45RA+CCR7+	14
	pT_{EFF}	CD45RA+CCR7-	13
	pT_{EM}	CD45RA-CCR7-	6, 11, 12, 15
Decidua	dT_N	CD45RA+CCR7+	14
	dT_{EFF}	CD45RA+CCR7-	10, 13
	dT_{EM}	CD45RA-CCR7- CD39-CD103-CD69-PD1-	11, 12
	dT_{RM1}	CD45RA-CCR7- CD39-CD103-CD69+PD1-	4
	dT_{RM2}	CD45RA-CCR7- CD39-CD103+CD69+PD1+	2
	dT_{RM3}	CD45RA-CCR7- CD39-CD103+CD69+	5, 7
	dT_{RM4}	CD45RA-CCR7- CD39+	1, 8, 9,15

Supplementary Table S2. Sorting of CD8+ T cell populations for functional testing

Antigen	Fluor	Clone	Company
CD4	BUV395	M-T477	BD Biosciences
	BV785	RPA-T4	Biolegend
CD56	BUV496	NCAM16.2	BD Biosciences
	PE	HCD56	Biolegend
CD3	BUV737	UCHT1	BD Biosciences
CD8	BUV805	RPA-T8	BD Biosciences
	APC	SKI	Biolegend
	BV510	HIT8a	Biolegend
CD103	BV421	Ber-ACT8	Biolegend
CD14	BV480	MOP9	BD Biosciences
	PerCP	HCD14	Biolegend
CD69	BV605	FN50	Biolegend
CD28	BV650	CD28.2	Biolegend
ICOS	BV711	C398.4A	Biolegend
CD27	BV750	O323	Biolegend
CD45	APC-Cy7	2D1	Biolegend
	SB550	2D1	Biolegend
CD25	PE	2A3	Biolegend
CD39	PE/Dzle594	A1	Biolegend
CD45RA	PE/Cy5	HI100	Biolegend
PD1	PE/Cy7	EH12.1	Biolegend
GITR	APC	DT5D3	Miltenyi Biotec
CCR7	A700	G043H7	Biolegend
	AF488	G043H7	Biolegend
TIGIT	PerCP/eF710	MBSA43	Biolegend
CTLA4	APC/Fire750	BNI3	Biolegend
GZMB	A488	351927	Invitrogen
GNLY	A647	DH2	Biolegend
Perforin	P.Blue	DG9	Biolegend
IFNɣ	APC	4S.B3	Biolegend
TNFα	P.Blue	MAb11	Biolegend
IL-2	PE	MQ1-17H12	Biolegend
CD107a	PerCP/CY5.5	H4A3	Biolegend
Caspase 3/7 detection reagent	Green	N/A	Invitrogen
HLA-A*02:01_HCMV_NLVPMVATV	FITC	N/A	Immudex,Denmark
HLA-A*02:01_HY_FIDSYICQV	PE	N/A	Immudex,Denmark

Supplementary Table 3. List of Antibody's used.
